# Constraining the tectonic evolution of rifted continental margins by U–Pb calcite dating

**DOI:** 10.1038/s41598-023-34649-z

**Published:** 2023-05-15

**Authors:** Åse Hestnes, Kerstin Drost, Tor O. Sømme, Deta Gasser, Thomas Scheiber, Henriette Linge, David Chew, Joachim Jacobs

**Affiliations:** 1 Department of Earth Science, University of Bergen, P.O.box 7803, 5020 Bergen, Norway; 2 Department of Geology, Trinity College Dublin, The University of Dublin, D02 PN40 Dublin 2, Norway; 3 Equinor, Martin Linges Vei 33, 1364 Fornebu, Norway; 4 Department of Environmental Sciences, Western Norway University of Applied Sciences, Campus Sogndal, P.O.Box 7030, 5020 Bergen, Norway; 5 Geological Survey of Norway, Torgarden, P.O.Box 6315, 7491 Trondheim, Norway

**Keywords:** Tectonics, Structural geology, Geodynamics

## Abstract

We employ U–Pb calcite dating of structurally-controlled fracture fills within crystalline Caledonian basement in western Norway to reveal subtle large-scale tectonic events that affected this rifted continental margin. The ages (15 in total) fall into four distinct groups with ages mainly ranging from latest Cretaceous to Pleistocene. (1) The three oldest (Triassic-Jurassic) ages refine the complex faulting history of a reactivated fault strand originated from the Caledonian collapse and broadly correlate with known rifting events offshore. (2) Two ages of ca. 90–80 Ma relate to lithospheric stretching and normal fault reactivation of a major ENE-WSW trending late Caledonian shear zone. (3) We correlate five ages between ca. 70 and 60 Ma with far-field effects and dynamic uplift related to the proto-Iceland mantle plume, the effect and extent of which is highly debated. (4) The five youngest ages (< 50 Ma) from distinct NE–SW trending faults are interpreted to represent several episodes of post-breakup fracture dilation, indicating a long-lived Cenozoic deformation history. Our new U–Pb data combined with structural and isotopic data show that much larger tracts of the uplifted continental margin of western Norway have been affected by far-field tectonic stresses than previously anticipated, with deformation continuing into the late Cenozoic.

## Introduction

The evolution of uplifted regions along rifted margins is the result of complex pre-, syn- and post-break up tectonic phases. When such uplifted margins are dominated by crystalline basement, the onshore rifting history is typically reconciled with the offshore tectono-sedimentary records by employing low-temperature thermochronology^1,2^. However, whereas offshore evidence often indicates protracted post-break up tectonic activity, the resolution of low-temperature thermochronology is often insufficient to accurately constrain the small amounts (1–2 km) of onshore exhumation that is found on many uplifted rifted margins. U–Pb carbonate dating of fracture-fill calcite is a modern approach that can yield high-resolution temporal constraints on such onshore rift-related exhumation along passive margins^3,4^.

In this study, we present the first successful U–Pb carbonate dating study of fracture-fill calcite from western Norway, which unravels pre-, syn- and post-breakup tectonic pulses spanning from the late Triassic to the Pleistocene. The geochronological data are complemented by isotopic data that characterise the fluids from which the calcite grew.

## Geological setting

**Figure 1:**
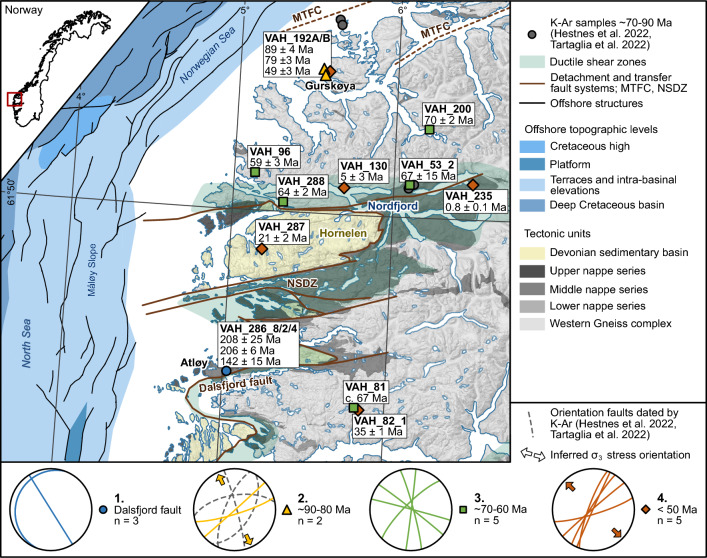
Simplified tectonic map of the study area with location of calcite samples. Map created with ArcGIS Pro v2.9.3 (www.esri.com). Inset of Norway with red box showing the study area. The fault and fracture orientation of the four age groups is represented on individual stereoplots; (1) Dalsfjord fault, (2) ~ 90–80 Ma fractures plotted together with orientation of faults of similar age determined by K–Ar fault gouge dating, (3) ~ 70–60 Ma fractures and faults with varying orientation, and (4) < 50 Ma NE-SW trending fractures. *MTFC* Møre-Trøndelag Fault Complex, *NSDZ* Nordfjord-Sogn Detachment Zone.

Western Norway is a classic example of a high elevation region bounding a rifted margin for which the tectonic evolution is debated^5–9^. The study area is dominated by the Western Gneiss Complex, which is overlain by the Caledonian nappe stack emplaced during the Silurian-Devonian Caledonian orogeny, as well as by younger Devonian basins^10^ (Fig. 1). Large-scale ductile to brittle detachment and transfer zones such as the Nordfjord-Sogn Detachment and the Møre-Trøndelag Fault Complex formed during initial collapse of the Caledonian orogen^11^. Major brittle Permian–Triassic and Late Jurassic-Early Cretaceous rift phases in the North Sea and the Norwegian Sea predated Late Cretaceous to Early Eocene rifting and final continental breakup^12^, with seafloor spreading starting in the Eocene at ca. 55 Ma^13^.

## Results

**Figure 2:**
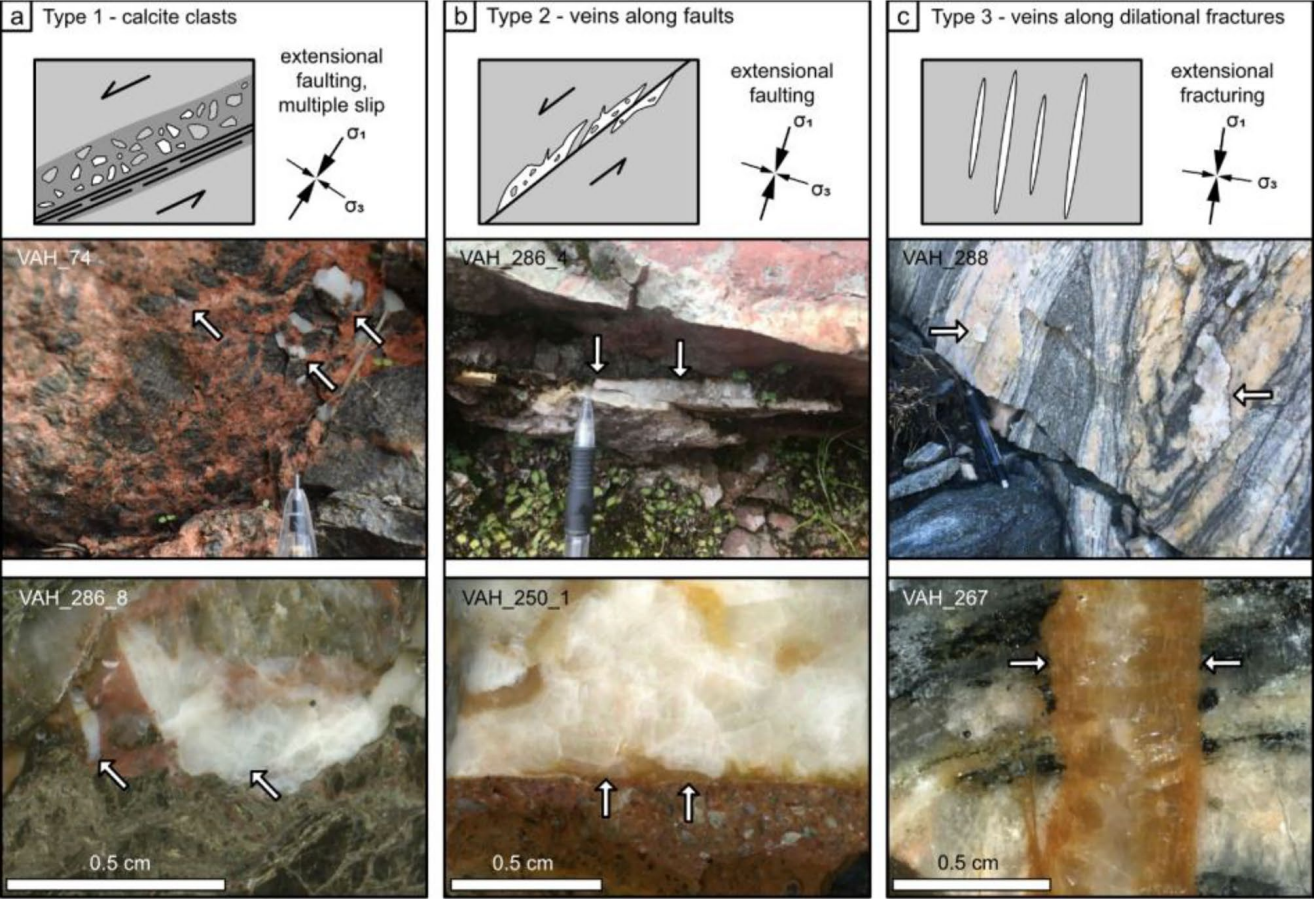
Schematic illustrations of calcite types and representative examples of calcite collected for U–Pb dating. Samples VAH_286_4, VAH_286_8 and VAH_288 were successfully dated while samples VAH_74, VAH_250_1 and VAH_267 did not yield sufficient U concentrations. (**a**) Type 1—calcite clasts or void filling incorporated into a cataclasite of a major fault zone (61.874396 N, 6.544759 E). (**b**) Type 2—calcite precipitated as veins along extensional fault slip surfaces (61.354915 N, 5.025077 E). (**c**) Type 3—calcite precipitated as veins in dilational fractures (61.862503 N, 5.314169 E). Black arrows indicate the local inferred paleostress orientation during formation of the faults and fractures. White arrows indicate calcite.

We collected 35 calcite samples from fractures and obtained 15 meaningful U–Pb ages from 14 different sites. The remaining 21 samples did not contain enough uranium (U) to extract reliable age information. All successfully dated calcites were collected from fracture planes lacking slickensides. Three different types of calcites have been dated (Fig. 2): type 1 represents calcite clasts or void filling incorporated in cataclasites within major brittle fault zones formed related to extensional faulting; type 2 represents calcite veins formed along distinct fault slip surfaces during extensional faulting, and type 3 represents simple dilational fractures not connected to brittle fault zones. We assume that the normal faults (type 1 and 2 calcites) formed at 30° to σ1, whereas the dilational fractures (type 3) formed parallel to σ1 and perpendicular to σ3. For detailed sample descriptions and U–Pb data, see Supplementary Material. Based on orientation, U–Pb age, calcite type and stable isotope composition, we assigned our results to four distinct groups, which in the following are described from the oldest to the youngest.

### Triassic to Cretaceous ages from the Dalsfjord fault

**Figure 3:**
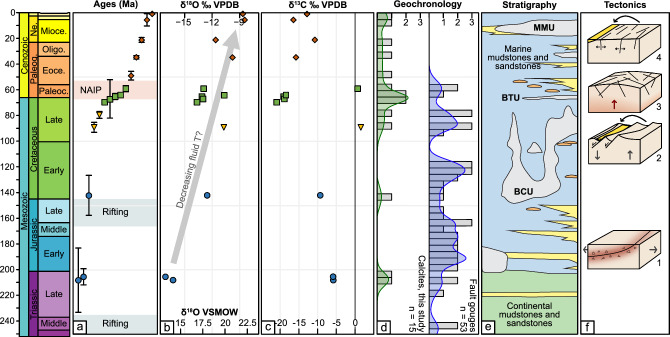
(**a**) U–Pb calcite ages with 2σ uncertainties. *NAIP* North Atlantic Igneous Province. (**b**) δ^18^O values, in VPDB (upper x-axis) and converted to VSMOW (lower x-axis)^48^. (**c**) δ^13^C values. Colour coding in (**a–c**) according to groups in Fig. 1. (**d**) Histogram (bin width 5 Ma) and Kernel distribution curve (bandwidth 5 Ma) of U–Pb calcite ages (green) and K–Ar fault gouge ages from western Norway (blue, modified from Hestnes et al.^18^). (**e**) Simplified stratigraphy from the northern North Sea. Modified from NPD (Norwegian Petroleum Directorate) lithostratigraphic chart, 2014. *MMU* Mid Miocene unconformity, *BTU* Base Tertiary unconformity, *BCU* Base Cretaceous unconformity. Green = continental mudstones; yellow = shallow-marine sandstones; orange = deep-marine sandstones; blue = marine mudstones. (**f**) Schematic sketches of the four interpreted tectonic episodes: (1) reactivation of the low-angle Dalsfjord fault, (2) unloading-loading and reactivation of the Møre-Trøndelag Fault Complex, (3) regional uplift due to the arrival of the Icelandic plume, and (4) pulses of local dilation and NE-SW trending fracture formation.

The three oldest obtained U–Pb calcite ages are from the Dalsfjord fault, separating the Western Gneiss Complex from Caledonian crystalline nappes^14^, through fault activity in the Late Permian-Early Triassic, Jurassic and Cretaceous^12,15,16^ (Fig. 1). The Dalsfjord fault contains distinct zones of green and red cataclasite as well as a prominent layer of fault gouge^12,15,17^ (see Supplementary Material 1). The two oldest calcite U–Pb ages come from a sub-rounded calcite clast or void filling within red cataclasite of the hanging wall (208.0 ± 25.0 Ma—VAH_286_8; Type 1; Fig. 3a, Table 1) and from a fracture plane cutting red cataclasite in the footwall (206.4 ± 6.2 Ma—VAH_286_2; Type 3; Fig. 3a, Table 1). A younger calcite was sampled on the fracture plane separating the red cataclasite from the gouge, giving an age of 142 ± 15 Ma (VAH_286_4; Type 2, Figs. 2b, 3a). For the older two calcites, the δ^18^O values are − 16.3‰ and − 17.09‰ (Fig. 3b), while the δ^13^C value is − 5.8‰ for both samples (Fig. 3c). The younger sample shows a δ^18^O value of − 12.5‰ (Fig. 3b) and a δ^13^C value of − 9.3‰ (Fig. 3c).

**Table 1:** Calcite sample information.

Sample ID	UTM32 Easting	UTM32 Northing	Elevation (m.a.s.l.)	dip dir./dip	δ^13^C (VPDB)	δ^18^O (VPDB)	U–Pb Age (Ma)	± 2 s	^207^Pb/^206^Pb_initial_	Analysis mode
VAH_286_8	287508	6808793	4	290/15	− 5.79‰	− 16.26‰	208	25	0.823 ± 0.006	Map, n = 40 (c. 30 s/20pixels)
VAH_286_2	287508	6808793	4	238/90	− 5.84‰	− 17.09‰	206.4	6.2	0.846 ± 0.011	Map, n = 140 (c. 30 s/20pixels)
VAH_286_4	287508	6808793	4	290/15	− 9.30‰	− 12.54‰	142	16	0.879 ± 0.026	Spots, n = 45
VAH_192B	319960	6907652	15	138/89	1.54‰	− 10.73‰	88.6	3.9	0.891 ± 0.005	Map, n = 75 (c. 30 s/25pixels)
VAH_192A	319960	6907652	15	163/81	1.83‰	− 6.06‰	79.4	2.7	0.856 ± 0.024	Map, n = 15 (c. 58 s/47pixels)
VAH_192A	319960	6907652	15	163/81	1.83‰	− 6.06‰	48.9	3.1	0.848 ± 0.021	Map, n = 15 (c. 58 s/47pixels)
VAH_200	354214	6888022	167	042/88	− 20.93‰	− 13.65‰	69.5	2.1	0.927 ± 0.030	Map, n = 34 (c. 63 s/50pixels)
VAH_53_2	347717	6869820	140	075/85	− 18.87‰	− 13.00‰	67	15	0.831 ± 0.023	Map, n = 17 (drawings, 59 to 403 s of signal)
VAH_81	330037	6796704	126	015/89	− 19.03‰	− 13.11‰	c. 67		–	Spots, n = 130
VAH_288	306151	6864364	8	097/89	− 18.45‰	− 10.69‰	64.0	2.0	0.874 ± 0.006	Map, n = 60 (c. 35 s/c. 20pixels)
VAH_96	296938	6874095	18	315/78	0.67‰	− 12.93‰	59.4	2.7	0.848 ± 0.0019	Spots, n = 77
VAH_82_1	330173	6796797	118	294/89	− 15.77‰	− 9.77‰	34.57	1.1	0.785 ± 0.027	Spots, n = 22
VAH_287	299163	6848868	25	302/84	− 10.72‰	− 11.62‰	21.1	1.5	0.7291 ± 0.0068	Map, n = 53 (c. 31 s/c. 22pixels)
VAH_130	326170	6868920	36	319/88	− 16.45‰	− 8.46‰	5.0	3.0	2.296 ± 0.053	Spots, n = 56
VAH_235	368469	6869810	60	290/80	− 12.79‰	− 8.65‰	0.83	0.10	0.9029 ± 0.0034	Spots, n = 78

### Late Cretaceous (90–80 Ma) ages

Two calcite samples collected from steep WSW-ENE-striking fracture planes in Precambrian gneisses of the Western Gneiss Complex on Gurskøya (Fig. 1) give U–Pb ages of 88.6 ± 3.9 Ma (VAH_192B; Type 3; Fig. 3a, Table 1) and 79.4 ± 2.7 Ma (VAH_192A, Type 3; Fig. 3a; Table 1). Their orientation is parallel to the strike of the Møre-Trøndelag Fault Complex further to the northeast^5^. For VAH_192B, the δ^18^O value is − 10.7‰ (Fig. 3b) and the δ^13^C value is 1.5‰ (Fig. 3c). For VAH_192A, the δ^18^O value is − 6.06‰ and the δ^13^C value is 1.83‰, but textural observations indicate that they most likely represent a mix between two calcite-forming episodes (see Supplementary Material 1), and the values are therefore excluded from figures and interpretations.

### Late Cretaceous-Paleogene (70–60 Ma) ages

Five samples, collected from steeply dipping fractures spread across the entire study area and having variable strike (Fig. 1), yield U–Pb ages of 69.5 ± 2.1 Ma (VAH_200; Type 3; Fig. 3a, Table 1), 67 ± 15 Ma (VAH_53_2; Type 2; Fig. 3a, Table 1), c. 67 Ma (VAH_81; Type 3; Fig. 3a, Table 1), 64.0 ± 2.0 Ma (VAH_288; Type 3, Figs. 2c, 3a, Table 1) and 59.4 ± 2.7 Ma (VAH_96; Type 3; Fig. 3a, Table 1). Three samples are from gneisses of the Western Gneiss Complex, whereas two are from the middle nappe series within the Nordfjord-Sogn Detachment (Fig. 1). Four of five samples show consistent δ^18^O values of − 13.7‰ to − 10.7‰ (Fig. 3b) and δ^13^C values of − 20.9‰ to − 18.5‰ (Fig. 3c). One sample (VAH_96, 59.0 ± 2.4 Ma) shows a δ^18^O value of − 12.9‰ (Fig. 3b) and a heavier δ^13^C value of 0.7‰ (Fig. 3c).

### Paleogene-Pleistocene (< 50 Ma) ages

Five samples, all collected from NE-SW striking fractures across the study area (Fig. 1), yield U–Pb ages of 48.9 ± 3.1 Ma (VAH_192A; Type 3; Fig. 3a, Table 1), 34.6 ± 1.1 Ma (VAH_82_1; Type 3; Fig. 3a, Table 1), 21.1 ± 1.5 Ma (VAH_287; Type 3, Fig. 3a, Table 1), 5.0 ± 3.0 Ma (VAH_130; Type 3; Fig. 3a, Table 1) and 0.83 ± 0.10 Ma (VAH_235; Type 3; Fig. 3a, Table 1). Two samples are from gneisses of the Western Gneiss Complex, two from the middle nappe series in the Nordfjord-Sogn Detachment, and one from the Hornelen Devonian basin (Fig. 1). The δ^18^O values vary from -11.7‰ to -8.5‰ (Fig. 3b) and the δ^13^C values vary from − 16.5 to − 10.6‰ (Fig. 3c).

## Discussion

Our new 15 U–Pb calcite ages are the first radiometric constraints of calcite-filled fractures from the onshore rifted margin of western Norway. They span across ~ 200 million years of tectonic evolution and correlate to some extent with well-known tectonic events but reveal also more subtle tectonic processes.

### The Dalsfjord fault: red cataclasite formation in the latest Triassic

The oldest three samples from the Dalsfjord fault provide further constraints on the faulting history of this important, complex fault zone. Palaeomagnetic, ^40^Ar/^39^Ar and K–Ar dating methods have earlier been used to constrain the activity along the Dalsfjord fault. Green cataclasite was interpreted to have formed at *c.* 260–248 Ma^15,16^ and red cataclasite at* c*. 150 Ma^15^. However, the 150 Ma paleomagnetic pole is poorly constrained^16^, and the ^40^Ar/^39^Ar ages of < 162 Ma are potentially disturbed by gouge formation, which is dated to *c.* 117–91 Ma^12^.

The two oldest calcite ages yield two possible interpretations for the age of the red cataclasite, both being older than the previously suggested age of *c.* 150 Ma^15^. In our preferred textural interpretation, the oldest calcite of 208 ± 25 Ma represents a calcite clast deformed within the cataclasite (Type 1) and the younger calcite of 206 ± Ma comes from a fracture cutting the cataclasite (Type 2). In this case, the two ages bracket the age of red cataclasite formation, which then must have occurred in the latest Triassic, representing a faulting phase not previously been detected (Fig. 3). Alternatively, if the age of 208 ± 25 Ma comes from a void-filling calcite post-dating cataclasite formation, then both ages represent minimum ages for red cataclasite formation, which then is constrained to have occurred in the Triassic between the 260–248 Ma green cataclasite and the 208–205 Ma calcite ages. The youngest calcite age142 ± 16 overlaps with the paleomagnetic age of ca. 150 Ma and represents renewed fault activity in the latest Jurassic—earliest Cretaceous, prior to gouge formation. The older ages of this study coincide with the observed increase in fault activity in western Norway prior to the Late Jurassic rift phase^12,18^ (Fig. 3d), whereas the younger age coincides with the prominent phase of rifting offshore in the Late Jurassic-Early Cretaceous^19^ (Fig. 3a,f).

### 90–80 Ma: Møre–Trøndelag fault complex reactivation

The two fractures on Gurskøya revealing Cretaceous calcite ages of 90–80 Ma strike parallel to the Møre-Trøndelag fault strands further northeast^20^. Apatite fission track data show cooling of the innermost block of the Møre-Trøndelag Fault Complex in the latest Cretaceous, related to top-NW-down normal faulting and associated footwall exhumation during NW-directed extension^5^. Four K–Ar fault gouge ages from faults with varying strike in the same region yield 90–70 Ma^18,21^ (stipled lines in stereoplot 2 in Fig. 1), temporally coinciding with the calcite U–Pb ages (Fig. 3d). The NNW-SSE trending σ_3_ inferred from the fracture orientations is compatible with the Cretaceous regional stress field affecting the mid-Norwegian passive margin at this time^18,21^. The timing of fracture formation corresponds to the renewed onset of rifting between Greenland and Norway at around 80 Ma, after a ~ 40 Ma period of quiescence^22^. This lithospheric extension left the crust flexed and weakened, and erosion induced unloading-loading along the margin has been interpreted to have caused Cretaceous top-NW-down normal reactivation of the Møre-Trøndelag fault system^5^ (Fig. 1). We interpret the 90–80 Ma calcite-filled fractures to be the product of the same process (Fig. 3f). The ages also coincide with pulses of deep-water turbidite deposition offshore^23^.

### 70–60 Ma: large-scale doming prior to North Atlantic break-up?

Five samples with ages between 70 and 60 Ma represent a regional period of fracture opening and calcite precipitation, pre-dating North Atlantic break-up. This time frame broadly coincides with the emplacement of the proto-Icelandic plume. It resulted in the onset of widespread volcanic activity from 63 to 62 Ma (North Atlantic Igneous Province or NAIP), the formation of regional unconformities offshore and dynamic uplift of off- and onshore regions during the Paleocene along the NW European margin^24–28^. The NAIP was active until Early Eocene times, ending *c.* 54 Ma ago^24,29^ (Fig. 3a). Different models have shown that the arrival of the proto-Icelandic plume could have caused uplift starting at c. 70 Ma and reaching a maximum of 0.1–0.6 km around 56–55 Ma^28,30^. Uplift of western Norway is supported by the presence of an up to 2 km thick clastic sedimentary wedge on the Måløy Slope offshore of the study area, which has been related to hinterland uplift^31,32^, and which overlaps in time with our fracture calcite ages (Fig. 1, 3e). We therefore interpret the 70–60 Ma calcite-filled fractures to represent onshore dynamic uplift affecting the NW Europe margins during the arrival of the proto-Icelandic plume (Fig. 3f). The variable orientation of the fracture surfaces in this group may indicate that fracture formation was related to large-scale domal uplift rather than the result of a regional unidirectional stress field (Fig. 1).

###  < 50 Ma: unravelling Eocene, Miocene, Pliocene and Pleistocene tectonic pulses

The five ages that are < 50 Ma were all obtained from NE-SW striking fractures (Fig. 1), and document continued tectonic activity throughout the entire Cenozoic (Fig. 3d). The offshore stratigraphy from the northern North Sea (Fig. 3e) shows an Oligocene phase of increased sediment input around 33–27 Ma, as well as a Mid Miocene unconformity from c. 25 to 8 Ma^33^. Different mechanisms have been suggested to explain these observations, such as long-term tectonic uplift due to far-field compression, episodic normal fault reactivation related to lithospheric flexure, and/or climatic variations including Quaternary glaciations, all potentially enhanced by unloading-loading mechanisms^34–38^. As a first order assumption, the fracture orientation is compatible with a NW–SE trending σ_3_ stress, indicating that these fractures might have formed in a NW–SE extensional or transtensional stress field from 50 Ma, similar to the inferred stress regime offshore from the Eocene to present^39^.

### Fluid sources

**Figure 4:**
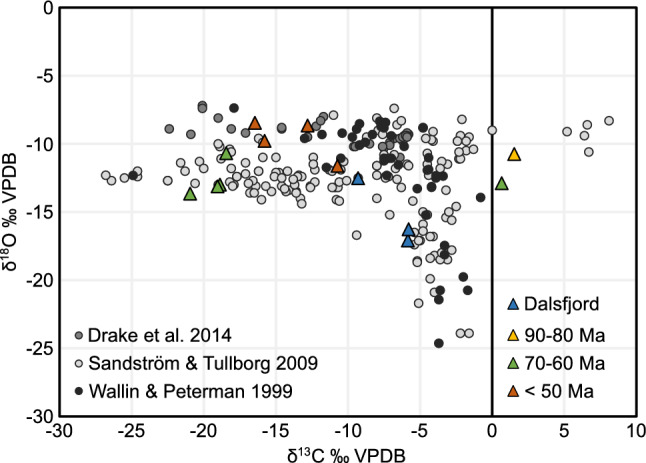
δ^13^C vs δ^18^O values of our samples (colour-coded according to Fig. 1d) and compared to data from the literature from similar geological settings.

The isotope analyses show δ^18^O values ranging from − 17.1 to − 8.5‰ and δ^13^C ranging from − 20.9 to − 5.8‰, with two exceptions showing slightly positive δ^13^C values (Figs. 3, 4). The generally depleted values of δ^18^O and δ^13^C indicate typical groundwater within crystalline basement as a water source, as demonstrated from other regions with a similar geological setting^40–42^ (Fig. 4). The general δ^18^O trend of getting heavier with younger ages (Fig. 3b), might indicate precipitation of calcite from fluids of a similar source under decreasing temperatures^40^, possibly indicating decreasing depth of crystallisation. The variably negative δ^13^C values point to the groundwater being influenced by dissolved HCO_3_^-^ from the oxidation of overlying organic material^43^, whereas the slightly positive δ^13^C values of the coastal samples VAH_192_B and VAH_96 (Fig. 1), point to a lack of overlying organic material and a possible influence of sea water.

## Conclusions

The first U–Pb calcite ages from fracture fills in basement rocks of western Norway demonstrate the potential of U–Pb calcite geochronology to reveal both large-scale regional and more subtle tectonic events along deeply eroded and uplifted continental margins. In particular, our data (1) reveal the potential influence of the proto-Icelandic plume in the region, leading to extensive fracturing with variable strike orientation, and (2) highlight the importance of ongoing post-break up tectonic activity throughout the entire Cenozoic. Our new U–Pb data combined with structural and isotopic data provide evidence that much larger crustal tracts of continental crust have been affected by far-field tectonic stresses than previously anticipated. These data are of prime importance for the understanding of rifted continental margins comprised of old basement, where the lack of younger sedimentary cover otherwise hampers constraining the regional tectonic events.

## Methods

### Laser ablation ICP-MS U–Pb calcite dating

Polished rock slabs in 25 mm diameter epoxy mounts were analysed for characteristic major and trace elements and for U and Pb isotopes using either a mapping approach or a spot ablation sampling strategy. Analyses were performed at the Department of Geology at Trinity College, Dublin using a Photon Machines Analyte Excite 193 nm ArF excimer laser ablation system coupled to an Agilent 7900 quadrupole ICP-MS.

We followed the general analytical and data processing routine for image-based U–Pb geochronology^44^ and specific details are given below in Supplementary Material 1 Table S1. In brief, laser sampling employed ablation of successive linear rasters that were compiled into element, elemental ratio and isotope ratio maps whereby one pixel represents one time-slice of the time-resolved signal. Characteristic major, minor and trace elements were measured along with U and Pb isotopes. Filtering of the data associated with the pixels in the maps was undertaken by applying a combination of specific geochemical criteria and/or manually drawn regions of interest to separate pixels from chemically and texturally different domains. The selected pixels were then pooled into ‘pseudo-analyses’ by using an empirical cumulative distribution function (ECDF) of a suitable channel (^238^U/^208^Pb or ^207^Pb/^235^U) and plotted on isochron diagrams. Details on the selection criteria and regions of interest are provided with the sample portraits of Supplementary material 1.

Spot analysis experiments were conducted in the conventional way. Downhole fractionation was negligible due to the large spot diameter and associated large width to depth ratio. Specific operating conditions and details on data processing are given in Supplementary Material 1 Table S2.

We use recommended uncertainty propagation^45^ with modifications^46^. The first uncertainty quoted in the tables (Supplementary Material 1) is a session-wide estimate including the data point uncertainty, uncertainty on weighted means of primary reference material ratios and their excess scatter. The second uncertainty additionally includes systematic uncertainties such as the uncertainty on the reference age of WC-1, uncertainty on the ^238^U decay constant and a laboratory-specific long-term reproducibility based on the results of the QC material (2%).

Ages were calculated using Isoplot 4.15. All U–Pb ages are from unanchored model 1 regressions in Tera Wasserburg (TW) plots (except VAH_130: unanchored regression in 86-TW space^47^). Uncertainties quoted in the main text and figures are 2σ and include systematic uncertainties.

### Stable isotope analyses

Stable isotope analyses were measured using a Finnigan MAT253 mass spectrometer coupled to a Kiel IV carbonate device at FARLAB at the Department of Earth Science, University of Bergen. Results are expressed as the average of the replicates and reported relative to Vienna Pee Dee Belemnite (VPDB), calibrated using NBS-19, and cross-checked with NBS-18. In addition, in-house standards CM12 and Riedel were also run. Long-term external precision (1σ SD) of the working standard (CM12) run in parallel with the samples is ≤ 0.08‰ and 0.03‰ for δ^18^O and δ^13^C, respectively, with sample sizes ranging between 15 and 100 µg. To assure reproducibility of the stable isotope analyses, 10 samples were duplicated 2, 3 or 4 times. The variability of the replicated samples was < 0.001–0.22‰ for δ^13^C and 0.001–2.8‰ for δ^18^O.

## Supplementary Information

Supplementary Information 1.

Supplementary Information 2.

## Data Availability

All data generated or analysed during this study are included in this published article (and its Supplementary Material files).
