# Supplementary material for: Constraining the tectonic evolution of rifted continental margins by U–Pb calcite dating
Source: Sci Rep. 2023 May 15;13:7876. doi: 10.1038/s41598-023-34649-z (PMC10185572; doi:10.1038/s41598-023-34649-z)
Supplement: Supplementary file 1 — Supplementary Information 1. [file 41598_2023_34649_MOESM1_ESM.pdf]

Supplementary material 1 – Description of calcite samples, including U-Pb age interpretation.

Supplement to the article: *Constraining the tectonic evolution of rifted continental margins by U-Pb calcite dating*

Åse Hestnes, Kerstin Drost, Tor O. Sømme, Deta Gasser, Thomas Scheiber, Henriette Linge, David Chew, Joachim Jacobs

Contents

Supplementary material 1 – Description of calcite samples, including U-Pb age interpretation. 1
- Sample portraits 2
- Overview Dalsfjord fault 2
- VAH_286_8 3
- VAH_286_2 4
- VAH_286_4 5
- VAH_192B 6
- VAH_192A 7
- VAH_200 9
- VAH_53_2 10
- VAH_81 11
- VAH_288 12
- VAH_96 13
- VAH_82_1 14
- VAH_287 15
- VAH_130 16
- VAH_235 17
- Table S1 - LA-ICP-MS operating conditions for mapping experiments 18
- Table S2 - LA-ICP-MS operating conditions for spot experiments 20
- References 22

Sample portraits

The following section provides a portrait of each analysed sample.

The structural data are given as dip direction/dip angle.

Imagery provided includes outcrop photograph, an image of the sample prepared for LA-ICP-MS analysis (polished 25mm diameter epoxy mount) with spot or map locations, and - in case of mapping experiments - an element map with the pixels used for age calculation shown in green.

Overview Dalsfjord fault

Three samples from the Dalsfjord fault were successfully dated: VAH_286_2, VAH_286_4 and VAH_286_8. Their structural position within the complex fault are shown in the sketch below (Fig. S1).

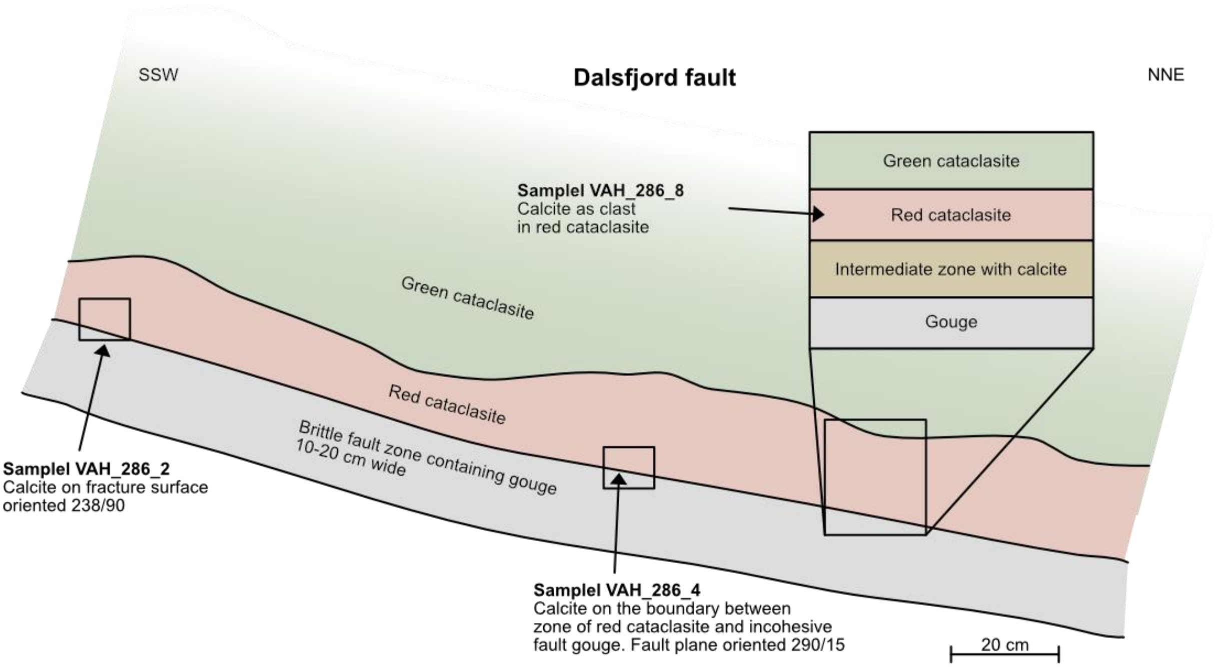

Figure S1 - Dalsfjord fault is part of the Nordfjord Sogn Detachment. At Atløy, the fault consists of green and red cataclasite in the hanging wall separated from a green cataclasite in the footwall by incohesive fault rock. The incohesive fault zone is 10-20 cm thick and contains gouge.

VAH_286_8

UTM32 Easting: 287508 **UTM32 Northing**: 6808793 **Elevation (m)**: 4

Locality description: Calcite from the Dalsfjord fault on Atløy (Fig. S1) Sample VAH_286_8 is from within the red cataclasite close to the boundary to the gouge zone of the fault (Fig. S1). It has subangular form and we interpret it to represent a clast deformed in the red cataclasite.

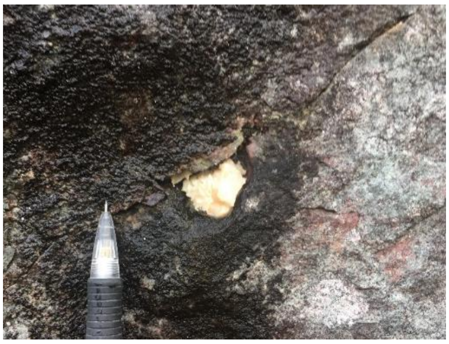

Figure S2 – calcite clast in red cataclasite.

Sample description: Calcite clast with irregular and weathered surface.

Fracture plane: 290/15

U-Pb analysis:
n=40 pseudo-analyses (c. 30s / 20 pixels each)
selection criteria: Ca_ppm_SQ_m43>350000; Th_U<2; Ce_ppm_SQ_m140<100

$^{207}Pb/^{206}Pb_{initial}$ = 0.823 ± 0.006
Lower intercept age: **208 ± 24 / 25 Ma**

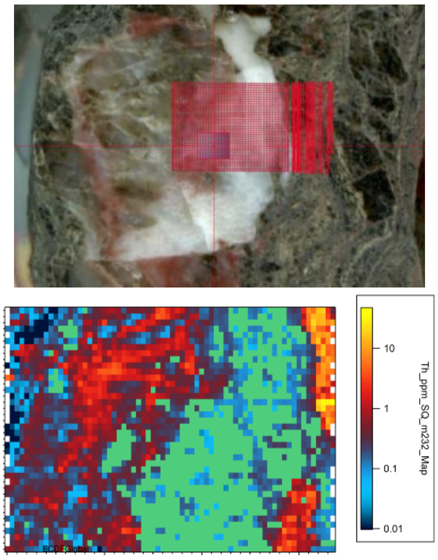

Figure S3 - Top: Location of mapped area (3120µm width by 2400µm height). Bottom: Thorium element map of the analysed area with pixels used for age calculation in green.

Remarks: Another mapping experiment and further spot analyses yield U-Pb data that are all identical within uncertainty.

Stable isotope data:
$\delta^{13}C$ (VPDB): -5.79 ‰
$\delta^{18}O$ (VPDB): -16.26 ‰

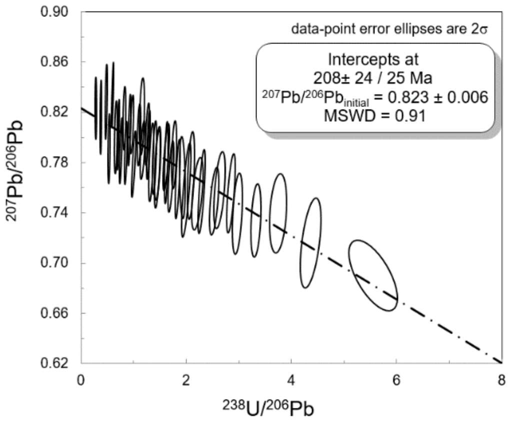

Figure S4 – Terra Wasserburg Concordia diagram.

VAH_286_2

UTM32 Easting: 287508 **UTM32 Northing**: 6808793 **Elevation (m)**: 4

Locality description: Calcite from the Dalsfjord fault on Atløy (Fig. S1). Sample VAH_286_2 contains fine-grained calcite collected on a small fracture surface within the red cataclasite.

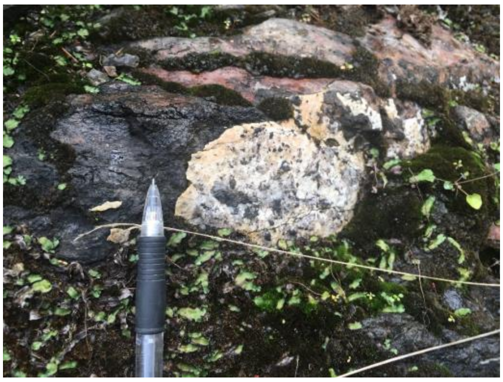

Figure S5 – Fracture plane cutting red cataclasite.

Sample description: Fine grained and thin fracture filling.

Fracture plane: 238/90

U-Pb analysis:
n=140 pseudo-analyses (c. 30s / 20 pixels each)
selection criteria: Th_ppm_SQ_m232<0.1; Ca_ppm_SQ_m43>350000; U238_CPS<190000; U238_CPS>4000

$^{207}Pb/^{206}Pb_{initial}$ = 0.846 ± 0.011
Lower intercept age: **206.4 ± 1.9 / 6.2 Ma**

Remarks: The low U centre of the vein appears to have a slightly more radiogenic initial Pb composition and was therefore excluded from age calculation.

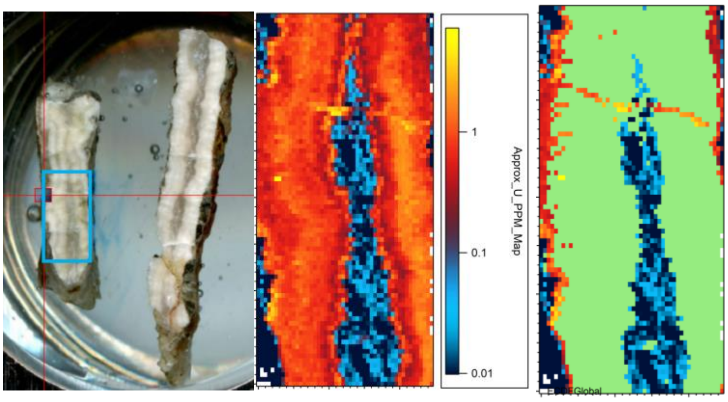

Figure S6 – Left: Location of mapped area (2310µm width by 4800µm height). Center: Semi-quantitative U element map. Right: Uranium map of analysed area with pixels used for age calculation shown in green.

Stable isotope data:
$\delta^{13}C$ (VPDB): -5.84 ‰
$\delta^{18}O$ (VPDB): -17.09 ‰

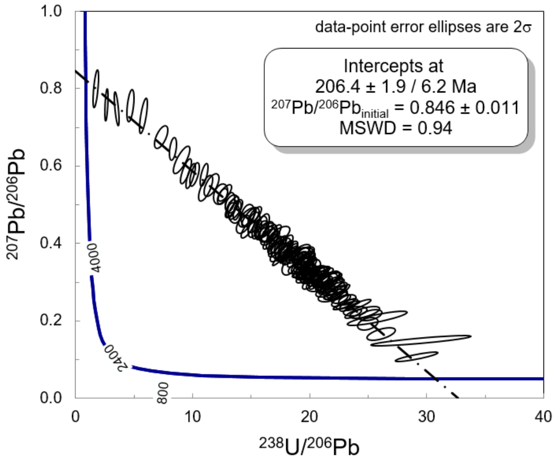

Figure S7 - Terra Wasserburg Concordia diagram.

VAH_286_4

UTM32 Easting: 287508 **UTM32 Northing**: 6808793 **Elevation (m)**: 4

Locality description: Calcite from the Dalsfjord fault on Atløy (Fig. S1). Sample VAH_286_4 was collected on the boundary between the red cataclasite and the gouge. The calcite mineralization was on a fault plane parallel to the gouge zone.

Sample description: cm-thick calcite in fault surface.

Fracture plane: 290/15

U-Pb analysis:
n=49 spot analyses, four of 49 rejected

$^{207}Pb/^{206}Pb_{initial} = 0.879 \pm 0.026$
Lower intercept age: **142 ± 15 / 16 Ma**

Remarks: Very low U concentration (<50ppb); except three spot analyses (50 to 100ppb) all of which are among the rejected data points.

Stable isotope data:
$\delta^{13}C$ (VPDB): -9.30 ‰
$\delta^{18}O$ (VPDB): -12.54 ‰

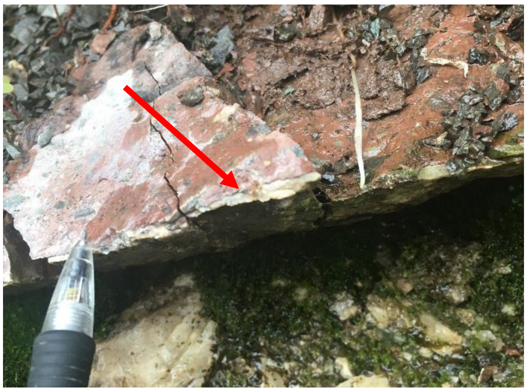

Figure S8- Calcite at the contact between red cataclasite above and gouge below.

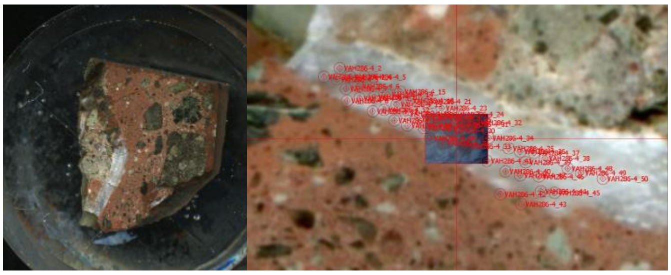

Figure S9 – Location of spot analyses.

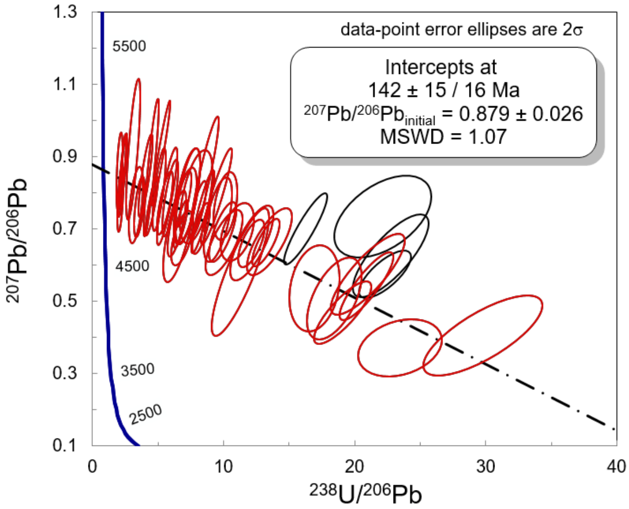

Figure S10 - Terra Wasserburg Concordia diagram. Four ellipses shown in black were excluded from calculations.

VAH_192B

UTM32 Easting: 319960 **UTM32 Northing**: 6907652 **Elevation (m)**: 15

Locality description: Calcite collected on Gurskøya on a fracture surface with patches of calcite.
The host rock at the sample location is a lens of mylonitic gneiss of Paleoproterozoic age surrounded by granitic orthogneisses of same age.

Sample description: Flaky and fine-grained calcite found together with pyrite mineralization.

Fracture plane: 138/89

U-Pb analysis:
n=75 pseudo-analyses (c. 30s / 25 pixels each)
selection criteria: Fe_ppm_SQ_m57<7000; Final207_206<1.5

$^{207}Pb/^{206}Pb_{initial}$ = 0.891 ± 0.005
Lower intercept age: **88.6 ± 2.9 / 3.9 Ma**

Remarks: The initial $^{207}Pb/^{206}Pb$ ratio is elevated when compared to Stacey and Kramer's (1975) terrestrial model Pb and likely reflects contributions from the surrounding Paleoproterozoic host rocks on the fluid composition.

Stable isotope data:
$\delta^{13}C$ (VPDB): 1.54 ‰
$\delta^{18}O$ (VPDB): -10.73 ‰

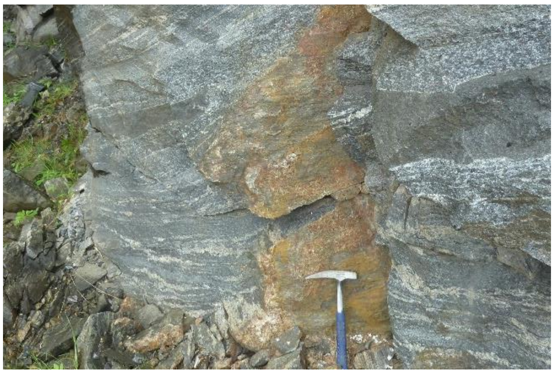

Figure S11- Brownish fracture plane with calcite.

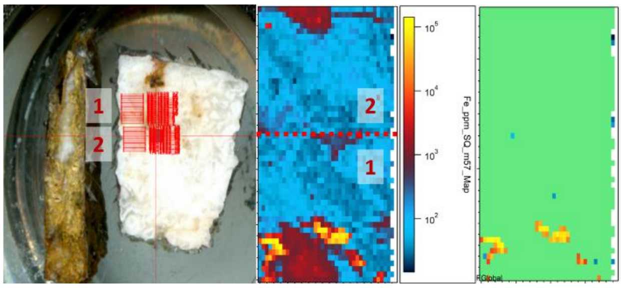

Figure S12 – Left: Location of mapped area (1530µm width by 3000µm height). Centre: Semi-quantitative Fe element map. Right: Iron map of analysed area with pixels used for age calculation shown in green.

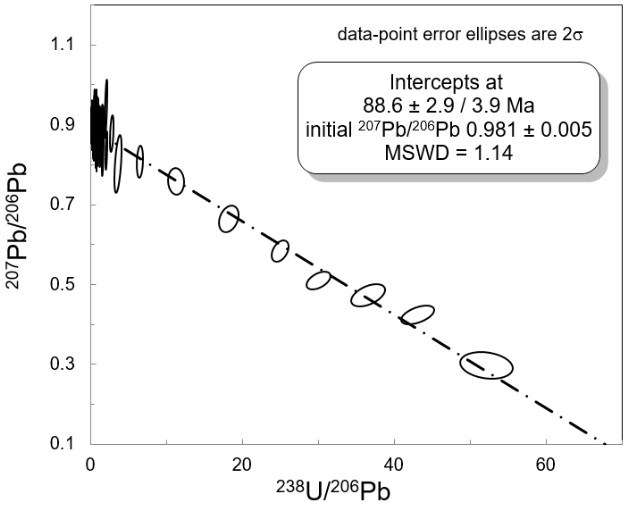

Figure S13 - Terra Wasserburg Concordia diagram.

VAH_192A

UTM32 Easting: 319960 **UTM32 Northing**: 6907652 **Elevation (m)**: 15

Locality description: Calcite collected on Gurskøya on a fracture surface containing patches of calcite. The host rock was highly fractures and the calcite contained clasts of the host rock as a result. Sample VAH_192A is collected on a fracture surface with a small angle to VAH_192B.
The host rock at the sample location is a lens of mylonitic gneiss of Paleoproterozoic age surrounded by granitic orthogneisses of same age.

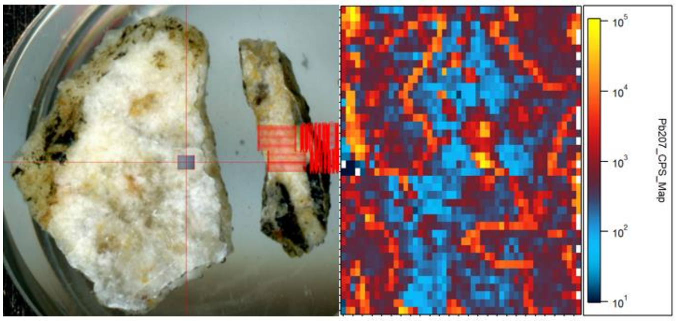

Figure S14 – Left: Location of mapped area (1920µm width by 2400µm height). Right: ^{207}Pb map.

Sample description: thin layer of calcite mineralization from fracture surface.

Fracture plane: 163/81

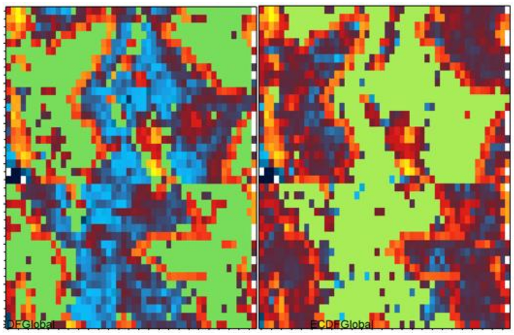

Figure S15 - Left: Pixels used for calculation of "rim" age are shown in green overlying the ^{207}Pb cps map. Right: Pixels used for calculation of "centre" age.

U-Pb analysis:
rim: n=15 pseudo-analyses (c. 58s / 47 pixels each)
selection criteria: Ca43_CPS>8M; Fe_ppm_m57<1500; V_ppm_SQ_m51 >0.2; all Pb masses_CPS>0

$^{207}Pb/^{206}Pb_{initial} = 0.856 \pm 0.024$
Lower intercept age: **79.4 ± 1.5 / 2.7 Ma**

centre: n=15 pseudo-analyses (c. 58s / 47 pixels each)
selection criteria: Ca43_CPS>8M; V_ppm_SQ_m51<0.1; all Pb masses_CPS>0

$^{207}Pb/^{206}Pb_{initial} = 0.848 \pm 0.021$
Lower intercept age: **48.9 ± 2.8 / 3.1 Ma**

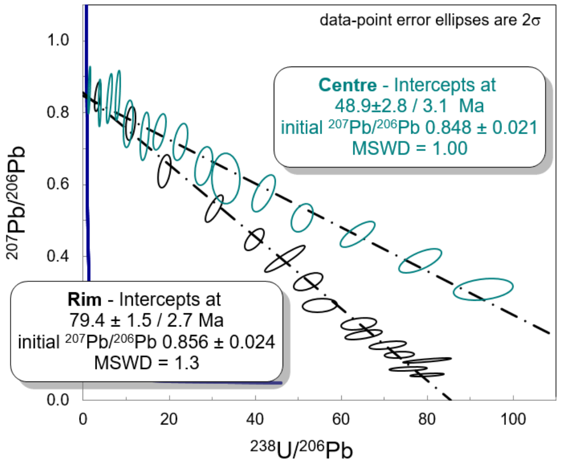

Figure S16 - Terra Wasserburg Concordia diagram.

Remarks: Since the centre age is younger than the rim age, we assume

the growth has been syntaxial (see Hilgers et al., 2001).

Stable isotope data:

$\delta^{13}C$ (VPDB): 1.83 ‰

$\delta\ ^{18}O$ (VPDB): -6.06‰

Since the material sampled for stable isotope analysis likely covers centre and rim age zones, we have excluded the isotopic results of the sample from the interpretations.

VAH_200

UTM32 Easting: 354214 **UTM32 Northing**: 6888022 **Elevation (m)**: 167

Locality description: Calcite collected along E39 in Austefjorden within a fault zone with several fault strands. Some gouge in the fault zone. The calcite crystallized on top of pale orange zeolite crystals and was found in the central area of a dense fracture network.
Located in Paleoproterozoic orthogneisses with close proximity to ultramafic lenses of Mesoproterozoic age.

Sample description: fracture of milky white calcite. Orange zeolite on the fracture wall.

Fracture plane: 042/88

U-Pb analysis:
n=34 pseudo-analyses (c. 63s / 50 pixels each)
selection criteria: Cu_ppm_SQ_m63 < 0.1; Th_ppm_m232<0.01; Pb206_CPS>0; Pb207_CPS>0; Pb208_CPS>0

$^{207}Pb/^{206}Pb_{initial}$ = 0.927 ± 0.030
Lower intercept age: **69.50 ± 0.76 / 2.1 Ma**

Remarks: The initial $^{207}Pb/^{206}Pb$ ratio is elevated when compared to Stacey and Kramer's (1975) terrestrial model Pb and likely reflects contributions to the fluid composition from the surrounding Proterozoic host rocks.

Stable isotope data:
$\delta^{13}C$ (VPDB): -20.93 ‰
$\delta^{18}O$ (VPDB): -13.65 ‰

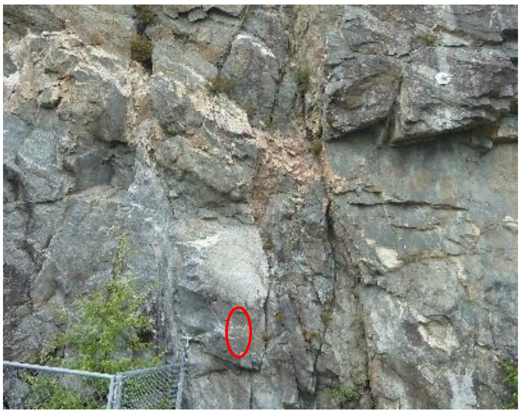

Figure S17- Brittle fault zone with several fault strands, calcite was collected on a near vertical strand in the lower part of the picture (red circle).

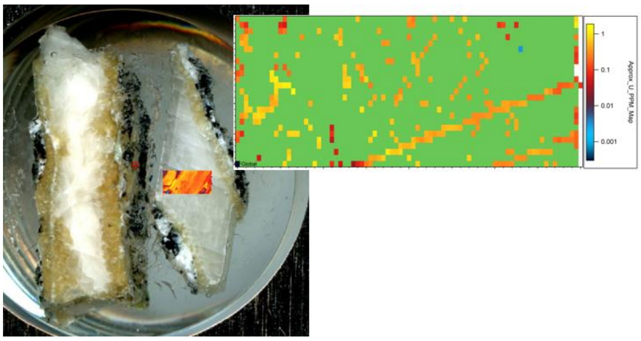

Figure S18 – Left: Semiquantitative U map showing the location of the mapped area (3060µm width by 1500µm height). Right: Pixels used for age calculation shown in green.

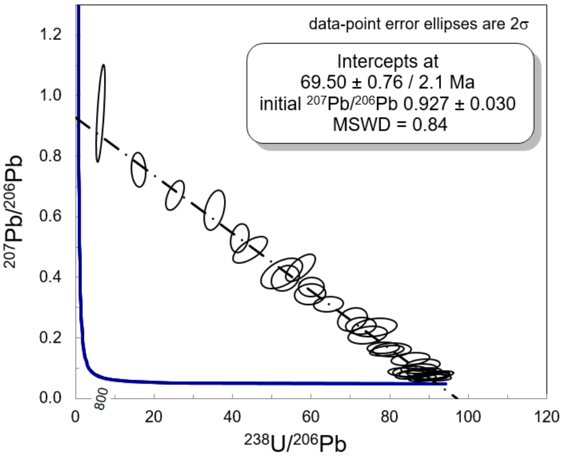

Figure S19 - Terra Wasserburg Concordia diagram.

VAH_53_2

UTM32 Easting: 347717 **UTM32 Northing**: 6869820 **Elevation (m)**: 140

Locality description: Calcite collected close to Hornindalsvatnet within a clay rich gouge fault zone. The calcite occurred as cm-scale crystals within the clay. The gouge shows a K-Ar age of 86 ± 2 Ma (Hestnes et al. 2022). There was also found calcite on the fault plane showing striated fibres (picture) (159-14), but this sample was not possible to date due to low U content. Host rock of the sample location is a Proterozoic banded gneiss, in places with Augen texture.

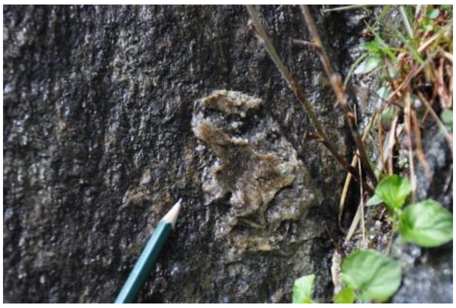

Figure S20- Calcite on fault plane.

Sample description: Clear cm scale calcite crystal.

Fracture plane: 075/85

U-Pb analysis:
n=17 manually drawn regions of interest (ROIs) (including 59 to 403 s of signal / 33 to 224 pixels)

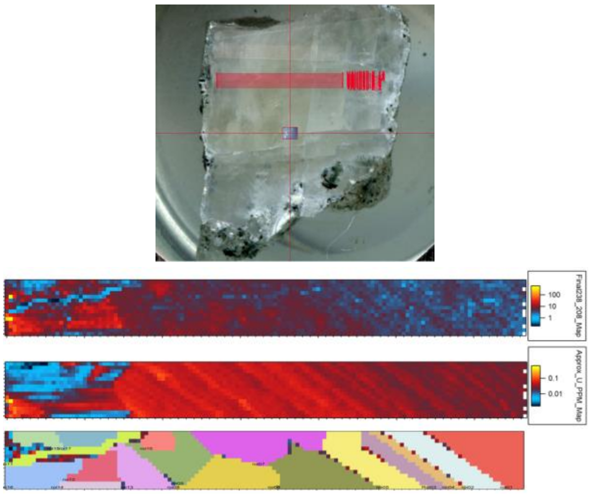

Figure S21 – Top: Location of mapped area (7380µm width by 900µm height). Middle: $^{238}U/^{208}Pb$ and semiquantitative U map. Bottom: Manually drawn ROIs (n=17).

$^{207}Pb/^{206}Pb_{initial}$ = 0.831 ± 0.023
Lower intercept age: **67 ± 15 / 15 Ma**

Remarks: Low U (4 to 46 ppb) and Pb (2 to 14 ppb) concentrations result in large uncertainties particularly on isotope ratios including ^{207}Pb and ^{208}Pb and thus on the calculated intercepts.

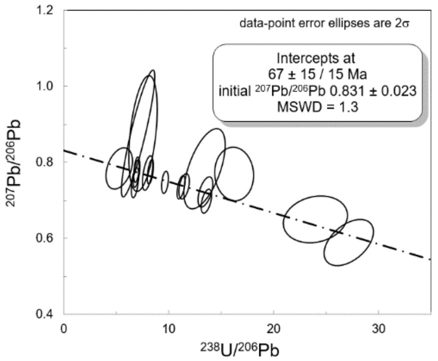

Figure S22 - Terra Wasserburg Concordia diagram. Ellipses correspond to the ROIs in Fig. S21.

Stable isotope data:
$\delta^{13}C$ (VPDB): -18.87 ‰
$\delta^{18}O$ (VPDB): -13.00 ‰

VAH_81

UTM32 Easting: 330037 **UTM32 Northing**: 6796704 **Elevation (m)**: 126

Locality description: Calcite collected along E39 in Ytredalen, from a 30 m long outcrop with subvertical foliation (123/81) and a parallel fault containing chlorite mineralization. The calcite-filled fracture set crosscuts the foliation and contain thin flakes of calcite crystals.
The host rock is a Proterozoic tonalitic to dioritic gneiss (WGR) in contact with a Proterozoic granitic orthogneiss, some places migmatitic and some places with Augen (WGR).

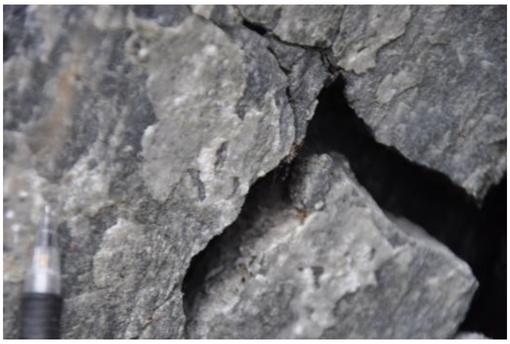

Figure S23- Calcite on fracture plane.

Sample description: Thin layer of mm-scale calcite crystals.

Fracture plane: 015/89

U-Pb analysis:
n=130 spot analyses

^{207}Pb/^{206}Pb$_{initial}$ and lower intercept are inconclusive due to an open U-Pb system

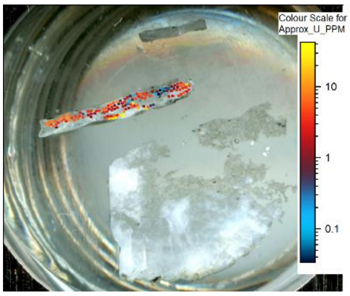

Figure S24 – Spot locations.

Remarks: An age of **c. 67Ma** seems likely for the main calcite forming event. However late U (and Pb?) mobility impede age calculation.
The U concentration is very variable (0.026 to 25.5 ppm) within the analysed area, and the initial ^{207}Pb/^{206}Pb appears to be highly radiogenic for the majority of the analyses.

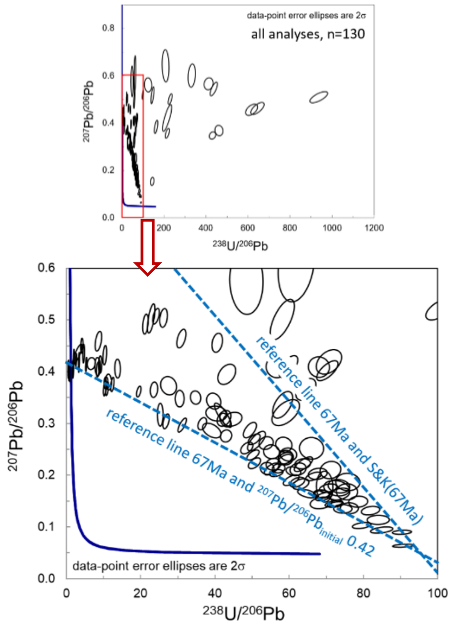

Figure S25 - Terra Wasserburg Concordia diagram. S&K is Stacey and Kramers (1975) terrestrial model lead.

Stable isotope data:
δ^{13}C (VPDB): -19.03 ‰
δ^{18}O (VPDB): -13.11 ‰

VAH_288

UTM32 Easting: 306151 **UTM32 Northing**: 6864364 **Elevation (m)**: 8

Locality description: Calcite collected close to Bortnen on a fracture surface containing flakes of calcite. The calcite was found as patches on a larger fracture plane (Fig. S26). The host rock at the locality is a Paleoproterozoic migmatitic orthogneiss.

Sample description: milky white calcite

Fracture plane: 097/89

U-Pb analysis:
n=60 pseudo-analyses (c. 35s / c. 20 pixels each)
selection criteria: Ca_ppm_SQ_m43>300000; U238_CPS>10; Rb_ppm_SQ_m85<0.1

$^{207}Pb/^{206}Pb_{initial}$ = 0.874 ± 0.006
Lower intercept age: **64.0 ± 1.0 / 2.0 Ma**

Remarks: -

Stable isotope data:
$\delta^{13}C$ (VPDB): -18.45 ‰
$\delta^{18}O$ (VPDB): -10.69 ‰

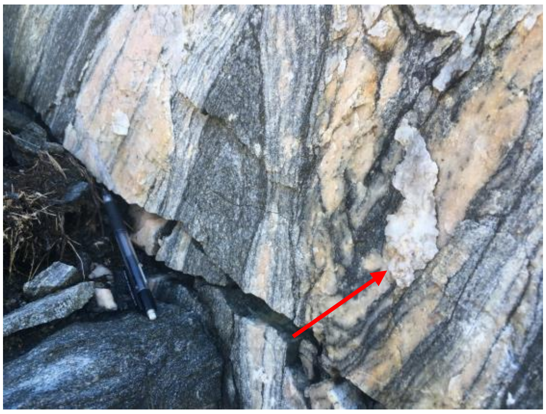

Figure S26- Calcite on fracture plane.

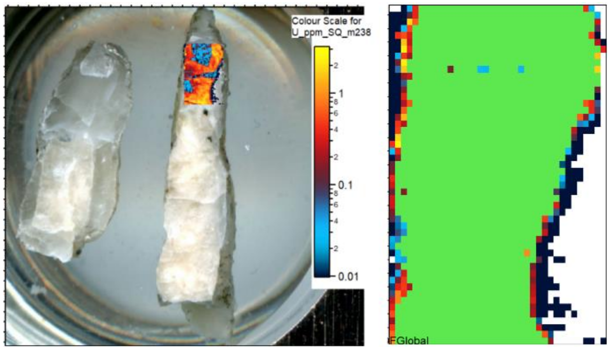

Figure S27 – Left: Semiquantitative U map showing the location of the mapped area (2130µm width by 3000µm height). Right: Pixels used for age calculation shown in green.

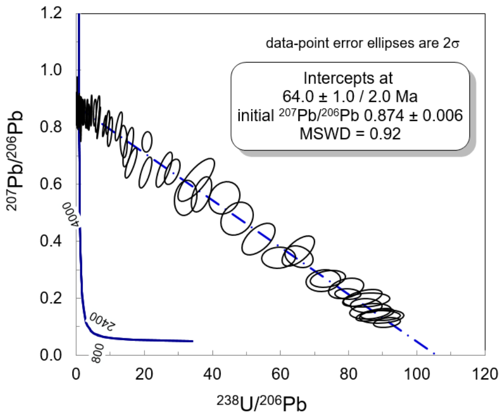

Figure S28 - Terra Wasserburg Concordia diagram.

VAH_96

UTM32 Easting: 296938 **UTM32 Northing**: 6874095 **Elevation (m)**: 18

Locality description: Calcite collected at Måløy on a fault surface containing chlorite, calcite and pyrite mineralization. The calcite and pyrite is found in patches on top of the fault surface. The chlorite shows slicken lines, while the calcite and pyrite precipitated on top lack these.
The host rock at the locality is Paleoproterozoic monzonitic gneiss.

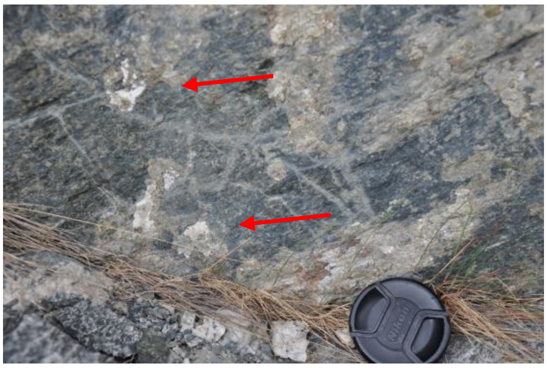

Figure S29- Calcite on fracture plane.

Sample description: Fine grained and milky white

Fracture plane: 315/78

U-Pb analysis:
A total of 117 spot analyses were made. Of those 40 analyses were rejected (three not calcite, one mixed phase, 36 from areas that were subject to resetting of the U-Pb system) and n=77 were used for calculation:

$^{207}Pb/^{206}Pb_{initial} = 0.848 \pm 0.0019$
Lower intercept age: 59.4 ± 2.0 / 2.7 Ma

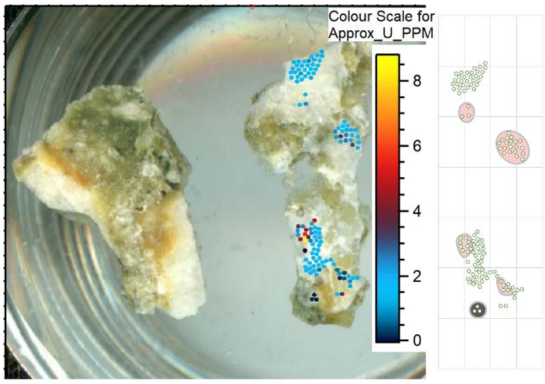

Figure S30 – Left: Spot locations. Right: Sketch of spot locations showing the used (green circles - no shading) and rejected analyses (red shading - open U-Pb system, grey - not calcite).

Remarks: Most part of the calcite has low µ. Certain areas within the sample appear to have not maintained a closed U-Pb system. This is suggested by a horizontal array of data points above the regression line.

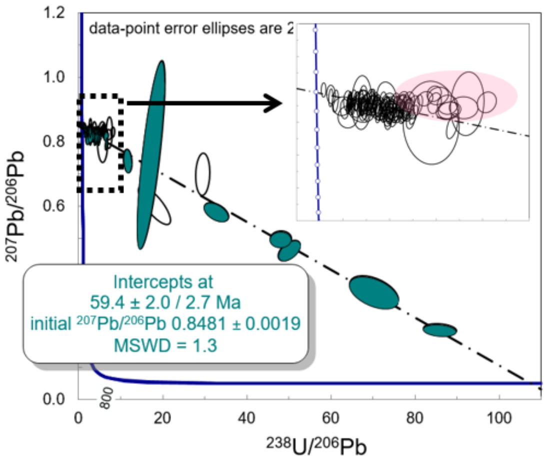

Figure S31 - Terra Wasserburg Concordia diagram showing all analyses (open ellipses) and those used for age calculation (teal ellipses).

Stable isotope data:
$\delta^{13}C$ (VPDB): 0.67 ‰
$\delta^{18}O$ (VPDB): -12.93 ‰

VAH_82_1

UTM32 Easting: 330173 **UTM32 Northing**: 6796797 **Elevation (m)**: 118

Locality description: Calcite collected along E39 in Ytredalen within a brittle fault zone. The main fault zone has thick chlorite mineralization. Calcite and quartz mineralizations are found on smaller surfaces, not directly on the main fault plane. Calcite crystals have grown on top of quartz. The host rock is highly fractured and occurs as clasts in the calcite.
The host rock is a Proterozoic tonalitic to dioritic gneiss (WGR) in contact with a Proterozoic granitic orthogneiss, some places migmatitic and some places with Augen.

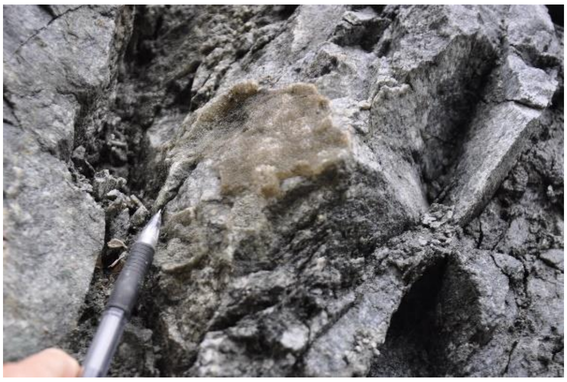

Figure S32- Calcite on fracture plane.

Sample description: mm scale clear calcite crystals

Fracture plane: 294/89

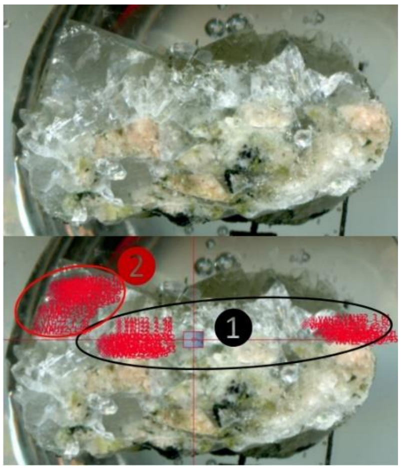

Figure S33 – Spot locations. Areas marked 1 and 2 correspond to discrete crystallisation stages.

U-Pb analysis:
area 1: n=22 spot analyses

$^{207}Pb/^{206}Pb_{initial}$ = 0.785 ± 0.027
Lower intercept age: **34.57 ± 0.65 / 1.1 Ma**

Remarks: Lead signals were extremely low due to relatively high μ and young age of the sample. Thus 23 of the 45 analyses run in area 1 had to be rejected.

area 2:
Remarks: The (sub-)concordant analyses in area 2 don't have enough Pb to retrieve a robust age. The six analyses with the highest U and Pb signals give an estimate for the timing of calcite crystallization in area 2.

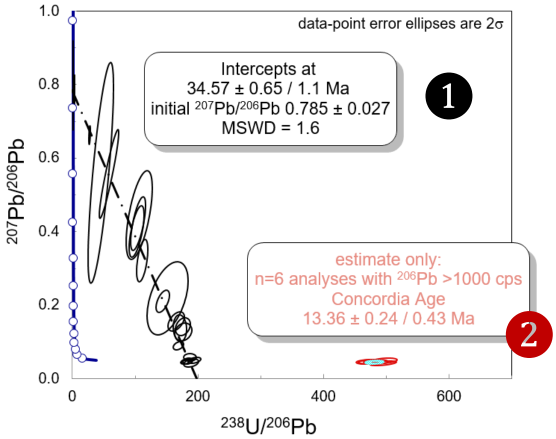

Figure S34 - Terra Wasserburg Concordia diagram. Data and statistics in black are for area 1, while the red ellipses give an estimate for area 2.

Stable isotope data:
$\delta^{13}C$ (VPDB): -15.77 ‰
$\delta^{18}O$ (VPDB): -9.77 ‰

VAH_287

UTM32 Easting: 299163 **UTM32 Northing**: 6848868 **Elevation (m)**: 25

Locality description: Calcite collected within the Hornelen Devonian sandstone basin on a large fracture surface containing large flakes of calcite.

Sample description: Fine-grained calcite. Appears white to pink on fracture surface, clear when polished.

Fracture plane: 302/84

U-Pb analysis:
n=53 pseudo-analyses (c. 31s / c. 22 pixels each)
selection criteria: Mg_ppm_SQ_m25 < 300; U238_CPS>100; Ca_ppm_SQ_m43>250000; Cu_ppm_SQ_m63<0.1; Pb207_CPS>50; Final207_206<1.5

$^{207}Pb/^{206}Pb_{initial}$ = 0.7291 ± 0.0068
Lower intercept age: **21.1 ± 1.3 / 1.5 Ma**

Remarks: -

Stable isotope data:
$\delta^{13}C$ (VPDB): -10.72 ‰
$\delta^{18}O$ (VPDB): -11.62 ‰

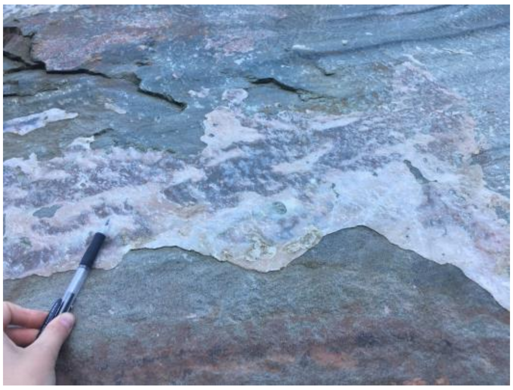

Figure S35- Picture taken of calcite on fracture plane.

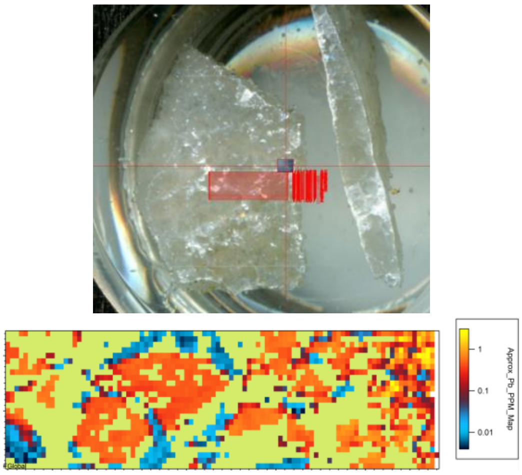

Figure S36 – Top: Location of the mapped area (4230µm width by 1500µm height). Bottom: Semi-quantitative Pb map with pixels used for age calculation shown in green.

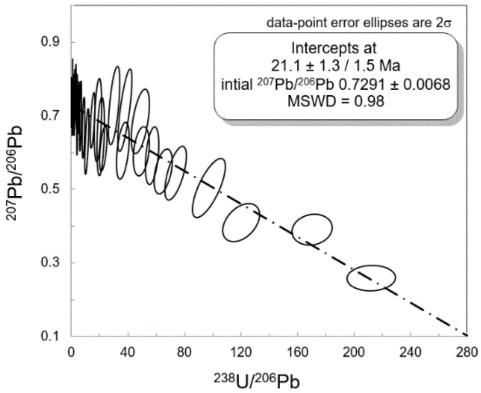

Figure S37 - Terra Wasserburg Concordia diagram.

VAH_130

UTM32 Easting: 326170 **UTM32 Northing**: 6868920 **Elevation (m)**: 36

Locality description: Calcite collected along Nordfjord from a several meter long fracture surface with a thick coating of epidote and chlorite. Flakes of yellowish and find grained calcite are found across the surface.
The host rock at the locality is a Paleoproterozoic orthogneiss of the WGR.

Sample description: Fine grained and yellowish calcite.

Fracture plane: 319/88

U-Pb analysis:
n=56 spot analyses

$^{208}Pb/^{206}Pb_{initial} = 2.296 \pm 0.053$
Lower intercept age: **5.0 ± 3.0 / 3.0 Ma**

Remarks: The ingrowth of radiogenic Pb is very limited due to the young age of the sample. Thus ^{207}Pb signals are extremely low. Signals of ^{208}Pb were slightly better, and we therefore opted for the date that was derived from regression of the data points in 86-TW space as suggested by Parrish et al. (2018).

Stable isotope data:
δ^{13}C (VPDB): -16.45 ‰
$\delta\, ^{18}$O (VPDB): -8.46 ‰

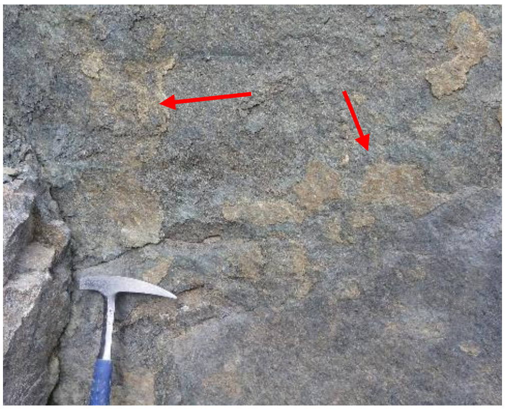

Figure S38- Yellowish calcite on fracture plane.

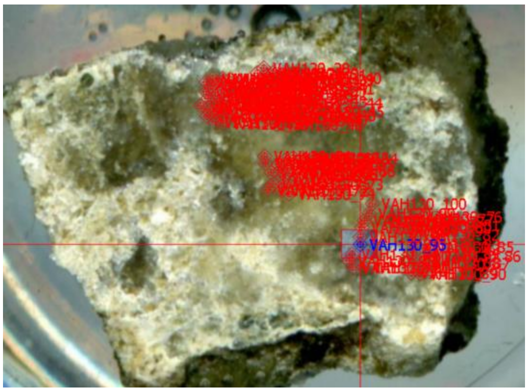

Figure S39 – Spot locations.

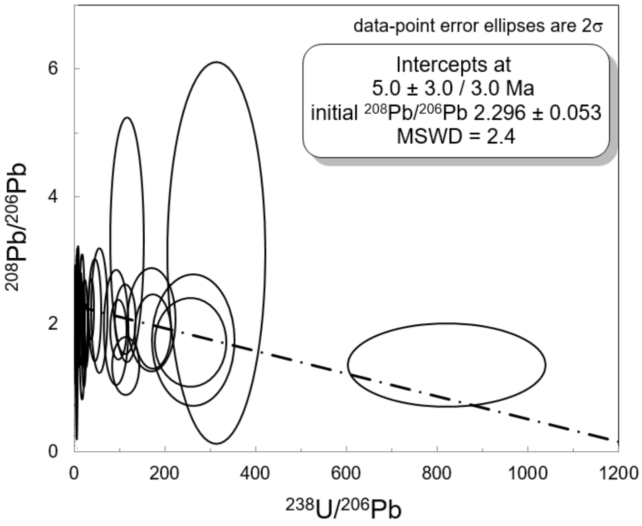

Figure S40 - 86-TW diagram (sensu Parrish et al., 2018).

VAH_235

UTM32 Easting: 368469 **UTM32 Northing**: 6869810 **Elevation (m)**: 60

Locality description: Calcite collected east of the Hornindalsvatnet from a fracture zone containing thick coatings of chlorite and minor epidote (Fig. S41). Milky white calcite has crystallized on top of the epidote and chlorite in thick clusters and thinner flakes.
The host-rock at the locality is a Paleoproterozoic Augen gneiss and banded gneiss dated 1625 ± 16 Ma (Mikkelsen, 2020).

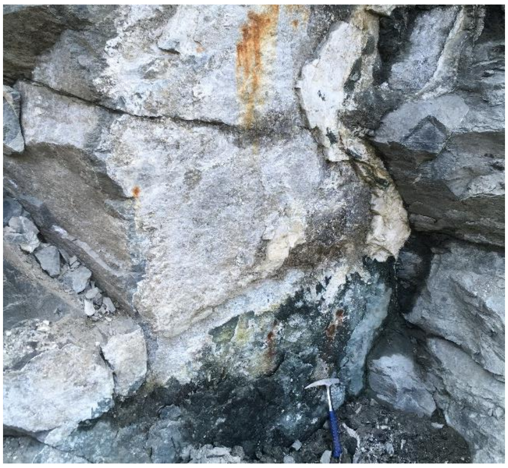

Figure S41- Calcite on fracture plane.

Sample description: The calcite is found as clear crystals and as milky white aggregates of fine-grained calcite. The milky white part of the calcite was chosen for further analyses.

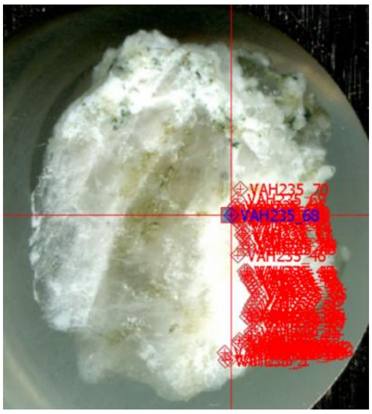

Figure S42 – Spot locations.

Fracture plane: 290/80

U-Pb analysis:
n=78 spot analyses

$^{207}Pb/^{206}Pb_{initial} = 0.9029 \pm 0.0034$
Lower intercept age: **0.83 ± 0.10 / 0.10 Ma**

Remarks: The initial $^{207}Pb/^{206}Pb$ ratio is elevated when compared to Stacey and Kramer's (1975) terrestrial model Pb and likely reflects contributions to the fluid composition from the surrounding Proterozoic host rocks.

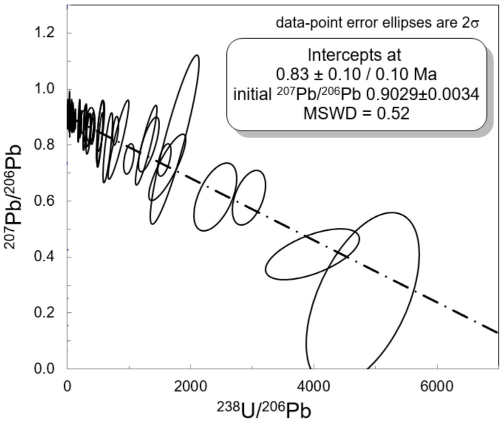

Figure S43 - Terra Wasserburg Concordia diagram.

Stable isotope data:
$\delta^{13}C$ (VPDB): -12.79 ‰
$\delta^{18}O$ (VPDB): -8.65 ‰

Table S1 - LA-ICP-MS operating conditions for mapping experiments

Sample type/mineral	calcite
Samples	(1) VAH 192A, VAH 192B, VAH 200 (transparent)<br>(2) VAH 286-2<br>(3) VAH 188, VAH 288, VAH 053-2<br>(4) VAH 286-3, VAH 286-1<br>(5) VA286-8<br>(6) VA286-8, VAH287, VAH288
Sample preparation	polished rock slab in 1 inch resin mount, 1µm polish to finish
Imaging	high-resolution scan of mount
**Laser ablation system**	
Make, Model & type	Teledyne/PhotonMachines Analyte Excite, 193nm, Excimer
Ablation cell & volume	HelEx II Active 2-volume cell; 100mm × 100mm sample area
Laser wavelength (nm)	193nm
Pulse width (ns)	<4ns
Fluence ($J.cm^{-2}$)	(1) 2.3 J/cm^2<br>(2-6) 2.2 J/cm^2
Repetition rate (Hz)	(1) 53 Hz<br>(2-6) 50 Hz
Spot size (um)	60 µm round
Sampling mode / pattern	linear rasters, 1 pass, 30 µm/sec scan speed
Carrier gas	100% He in the cell (0.40 l/min), Ar carrier gas and N_2 added at ARIS adaptor
Ablation duration (secs)	(1) NIST614 21 x 64s, WC-1: 28 x 26s, DBT: 28 x 62s, VAH192A 40 x 64s, VAH192B 50 x 51s, VAH200 25 x 102s<br>(2) NIST614 18 x 65s, WC-1: 18 x 37s, DBT: 18 x 62s, VAH286-2 80 x 77s<br>(3) NIST614 24 x 66s, WC-1: 24 x 37s, DBT: 24 x 62s, VAH 188 36 x 81s, VAH 288 50 x 71s, VAH 053-2 15 x 246s<br>(4) NIST614 18 x 63s, WC-1: 18 x 36s, DBT: 18 x 66s, VAH 286-1: 12 x 406s, VAH 286-3: 21 x 185s<br>(5) NIST614 16 x 64s, WC-1: 16 x 32s, DBT: 16 x 65s, VAH 286-8: 40 x 104s<br>(6) NIST614 18 x 62s, WC-1: 18 x 35s, DBT: 18 x 65s, VAH 286-8: 20 x 154s, VAH287 25 x 141s, VAH288 40x66 s
Cell carrier gas flow (l/min)	(1) 0.25 l/min in the cell and 0.15 l/min in the cup<br>(2, 3) 0.32/0.08 l/min<br>(4) 0.28/0.12 l/min<br>(5) 0.3/0.1 l/min<br>(6) 0.275/0.125 l/min
**ICP-MS Instrument**	
Make, Model & type	Agilent 7900 quadrupole ICP-MS
Sample introduction	Ablation aerosol via ARIS
RF power (W)	1550W

Carrier gas flow (l/min)	(1) 0.69 l/min Ar<br>(2) 0.60 l/min Ar<br>(3, 4) 0.65 l/min Ar<br>(5) 0.75 l/min Ar<br>(6) 0.70 l/min Ar
Detection system	Dual-mode discrete dynode electron multiplier
Masses measured and [Integration time per peak (ms)]	**(1, 2, 5)** 25 [1.5], 43 [3], 51 [2], 55 [1.5], 57 [1.5], 63 [2], 66 [2], 71 [2], 85 [2], 88 [1.5], 137 [2], 140 [2], 202 [1], 204 [1], 206 [32], 207 [69], 208 [32], 232 [20], 238 [32]<br>**(3, 4)** 25 [1.5], 43 [3], 51 [2], 55 [1.5], 57 [1.5], 63 [2], 66 [2], 71 [2], 85 [2], 88 [1.5], 137 [2], 140 [2], 202 [1], 204 [1], 206 [40], 207 [95], 208 [40], 232 [20], 238 [40]<br>**(6)** 25 [1.5], 43 [3], 51 [2], 55 [1.5], 57 [1.5], 63 [2], 66 [2], 71 [2], 85 [2], 88 [1.5], 137 [2], 140 [2], 202 [1], 204 [1], 206 [30], 207 [65], 208 [30], 232 [20], 238 [30]
Total integration time per reading (secs)	(1) 250ms / 1250 ms after averaging<br>(2, 5) 250ms / 1500 ms after averaging<br>(3) 300ms / 1800 ms after averaging<br>(4) 300ms / 1500 after averaging<br>(6) 240ms / 1440 ms after averaging
Sensitivity / Efficiency (%, element)	0.02% U
IC Dead time (ns)	38ns
**Data Processing**	
Gas blank	≥13 s on-peak zero subtracted
Calibration strategy	NIST614 as primary reference material, WC-1 carbonate standard for matrix matching of $^{206}Pb/^{238}U$, DBT carbonate for QC
Reference Material info	NIST614 (concentration data Jochum et al., 2011; Pb isotopes Woodhead and Hergt, 2001)<br>WC-1 (Roberts et al., 2017)<br>DBT (Hill et al., 2016)
Data processing package used / Correction for LIEF	Iolite V3.6 & Monocle & in-house spreadsheet; no LIEF correction for linear rasters
Normalization and age calculation	standard bracketing; Iolite Data Reduction Scheme VizualAge_UcomPbine (Chew et al. 2014; based on U-Pb Geochronology DRS of Paton et al., 2010 and VizualAge DRS of Petrus and Kamber, 2012) is used to correct for down hole fractionation and drift and to normalize to primary reference material. Downhole fractionation for linear rasters is modelled using a linear correction (y=a+bx) with zero slope (b=0). U/Pb ages and initial Pb compositions are calculated using Isoplot v4.15 (Ludwig, 2012).
Common-Pb correction, composition and uncertainty	Unanchored regression in Tera-Wasserburg, isochron and 86TW plots, respectively. All model 1.<br>Except WC-1: Anchored regression in TW using an initial $^{207}Pb/^{206}Pb$ of 0.85±0.04 (Roberts et al., 2017) to receive a non-matrix-matched lower intercept age, the corresponding ratio of which is used to calculate the matrix-dependent factor for correction of $^{206}Pb/^{238}U$ ratios of QC and unknowns

Uncertainty level & propagation	Ratios and ages are quoted at 2s. Uncertainty propagation was carried out according to the recommendations of Horstwood et al. (2016) and Roberts et al. (2020). The first uncertainty quoted is a session wide estimate including the data point uncertainty, uncertainty on weighted means of primary reference material ratios and their excess scatter (if applicable). The second uncertainty quoted additionally includes systematic uncertainties
Quality control / Validation	*DBT (Hill et al., 2016: 64.04 ± 0.67 Ma / 0.738 ± 0.010)*<br>(1) Lower Intercept Age = 64.7 ± 1.2/2.2 Ma, $^{207}Pb/^{206}Pb_{initial}$ = 0.698 ± 0.024 (2s, MSWD = 0.76)<br>(2) Lower Intercept Age = 64.96 ± 0.82/2.0 Ma, $^{207}Pb/^{206}Pb_{initial}$ = 0.701 ± 0.014 (2s, MSWD = 1.1)<br>(3) Lower Intercept Age = 63.72 ± 0.93/2.0 Ma, $^{207}Pb/^{206}Pb_{initial}$ = 0.704 ± 0.020 (2s, MSWD = 0.84)<br>(4) Lower Intercept Age = 65.0 ± 1.4/2.3 Ma, $^{207}Pb/^{206}Pb_{initial}$ = 0.702 ± 0.030 (2s, MSWD = 0.56)<br>(5) Lower Intercept Age = 64. 2 ± 1.0/2.0 Ma, $^{207}Pb/^{206}Pb_{initial}$ = 0.704 ± 0.022 (2s, MSWD = 0.65)<br>(6) Lower Intercept Age = 64.3 ± 1.8/2.7 Ma, $^{207}Pb/^{206}Pb_{initial}$ = 0.706 ± 0.030 (2s, MSWD = 0.21)
**Other information**	All samples were cleaned with ethanol followed by sonication in DIW. Potentially remaining surface contamination was removed during a preablation of all ablated sites. Detailed information on the general analytical protocol and data processing is given in Drost et al. (2018).

Table S2 - LA-ICP-MS operating conditions for spot experiments

Sample type/mineral	calcite
Samples	01.12.2020 - VAH 082<br>02.12.2020 - VAH 081<br>14.12.2020 - VAH 235, VAH 130, VAH 286-4<br>22.12.2020 - VAH 096<br>07.01.2021 - VAH 287, VAH 286-4, VAH 286-8
Sample preparation	polished rock slab in 1 inch resin mount, 1µm polish to finish
Imaging	high-resolution scan of mount
**Laser ablation system**	
Make, Model & type	Teledyne/PhotonMachines Analyte Excite, 193nm, Excimer
Ablation cell & volume	HelEx II Active 2-volume cell; 100mm × 100mm sample area
Laser wavelength (nm)	193nm
Pulse width (ns)	<4ns
Fluence ($J.cm^{-2}$)	2.3 J/cm^2
Repetition rate (Hz)	11 Hz
Spot size (um)	160 µm round
Sampling mode / pattern	spots

Carrier gas	100% He in the cell (c. 0.40 l/min), Ar carrier gas and N_2 added at ARIS adaptor
Ablation duration (secs)	385 shots (35s)<br>except 01.12.2020: 440 shots (40s) and 14.12.2020: 285 shots (26s)
Cell carrier gas flow (l/min)	optimized daily, 0.23 to 0.32 l/min in the cell and 0.08 to 0.15 l/min in the cup
**ICP-MS Instrument**	
Make, Model & type	Agilent 7900 quadrupole ICP-MS
Sample introduction	Ablation aerosol via ARIS (1mm ID PEEK tubing) and variable volume mixing device for signal smoothing
RF power (W)	1550W
Carrier gas flow	optimized daily: 0.70 to 0.90 l/min Ar
Detection system	Dual-mode discrete dynode electron multiplier
Masses measured and [Integration time per peak (ms)]	all: 25 [1.5], 43 [3], 51 [2], 55 [1.5], 57 [1.5], 85 [2], 88 [1.5], 140 [2], 202 [1], 204 [1], 206 [50], 207 [125], 208 [50], 232 [25], 238 [50]<br>except 01.12.2020: 25 [1.5], 43 [3], 51 [2], 55 [1.5], 57 [1.5], 85 [2], 88 [1.5], 140 [2], 202 [1], 204 [1], 206 [43], 207 [100], 208 [43], 232 [21], 238 [43]
Total integration time per reading	350ms, except 01.12.2020: 300ms
Sensitivity / Efficiency (%, element)	0.02% U
IC Dead time (ns)	38ns
**Data Processing**	
Gas blank	≥15 s on-peak zero subtracted
Calibration strategy	NIST614 as primary reference material, WC-1 carbonate standard for matrix matching of $^{206}Pb/^{238}U$, DBT carbonate for QC
Reference Material info	NIST614 (concentration data Jochum et al., 2011; Pb isotopes Woodhead and Hergt, 2001)<br>WC-1 (Roberts et al., 2017)<br>DBT (Hill et al., 2016)
Data processing package used	Iolite V3.6 & in-house spreadsheet
Normalization and age calculation	standard bracketing; Iolite Data Reduction Scheme VizualAge_UcomPbine (Chew et al. 2014; based on U-Pb Geochronology DRS of Paton et al., 2010 and VizualAge DRS of Petrus and Kamber, 2012) is used to correct for down hole fractionation and drift and to normalize to primary reference material. Downhole fractionation is modelled using a linear correction (y=a+bx). U/Pb ages and initial Pb compositions are calculated using Isoplot v4.15 (Ludwig, 2012).
Common-Pb correction, composition and uncertainty	Unanchored regression in Tera-Wasserburg, isochron and 86TW plots, respectively. All model 1.<br>Except WC-1: Anchored regression in TW using an initial $^{207}Pb/^{206}Pb$ of 0.85±0.04 (Roberts et al., 2017) to receive a non-matrix-matched lower intercept age, the corresponding ratio of which is used to calculate the matrix-dependent factor for correction of $^{206}Pb/^{238}U$ ratios of QC and unknowns
Uncertainty level & propagation	Ratios and ages are quoted at 2s. Uncertainty propagation is largely following the recommendations of Horstwood et al. (2016) and

	Roberts et al. (2020). The first uncertainty quoted is a session wide estimate including the data point uncertainty, uncertainty on weighted means of primary reference material ratios and their excess scatter (if applicable). The second uncertainty quoted additionally includes systematic uncertainties.
Quality control / Validation	*DBT (Hill et al., 2016: 64.04 ± 0.67 Ma / 0.738 ± 0.010)*<br>01.12.2020: Lower Intercept Age = 65.6 ± 1.0/2.0 Ma, $^{207}Pb/^{206}Pb_{initial}$ = 0.716 ± 0.016 (2s, MSWD = 0.78)<br>02.12.2020: Lower Intercept Age = 63.8 ± 3.1/3.7 Ma, $^{207}Pb/^{206}Pb_{initial}$ = 0.700 ± 0.068 (2s, MSWD = 1.14)<br>14.12.2020: Lower Intercept Age = 64.6 ± 2.4/3.1 Ma, $^{207}Pb/^{206}Pb_{initial}$ = 0.683 ± 0.044 (2s, MSWD = 0.94)<br>22.12.2020: Lower Intercept Age = 64.2 ± 2.3/3.0 Ma, $^{207}Pb/^{206}Pb_{initial}$ = 0.718 ± 0.048 (2s, MSWD = 1.11)<br>07.01.2021: Lower Intercept Age = 64.2 ± 4.2/4.5 Ma, $^{207}Pb/^{206}Pb_{initial}$ = 0.696 ± 0.089 (95% conf., MSWD = 1.3)
**Other information**	All samples were cleaned with ethanol followed by sonication in DIW. Potentially remaining surface contamination was removed during a preablation of all ablated sites (20 shots, 190µm spot diameter). Detailed information on the general analytical protocol and data processing is given in Drost et al. (2018).

References

Chew, D. M., Petrus, J. A., and Kamber, B. S., 2014, U–Pb LA–ICPMS dating using accessory mineral standards with variable common Pb: Chemical Geology, v. 363, p. 185-199, https://doi.org/10.1016/j.chemgeo.2013.11.006

Drost, K., Chew, D., Petrus, J. A., Scholze, F., Woodhead, J. D., Schneider, J. W., and Harper, D. A. T., 2018, An Image Mapping Approach to U-Pb LA-ICP-MS Carbonate Dating and Applications to Direct Dating of Carbonate Sedimentation: Geochemistry, Geophysics, Geosystems, v. 19, no. 12, p. 4631-4648, https://doi.org/10.1029/2018GC007850

Hilgers, C., Koehn, D., Bons, P.., and Urai, J.., 2001, Development of crystal morphology during unitaxial growth in a progressively widening vein: II. Numerical simulations of the evolution of antitaxial fibrous veins: Journal of Structural Geology, v. 23, p. 873–885, doi:10.1016/S0191-8141(00)00160-7.

Hill, C. A., Polyak, V. J., Asmerom, Y., and P. Provencio, P., 2016, Constraints on a Late Cretaceous uplift, denudation, and incision of the Grand Canyon region, southwestern Colorado Plateau, USA, from U-Pb dating of lacustrine limestone: Tectonics, v. 35, no. 4, p. 896-906, https://doi.org/10.1002/2016TC004166

Horstwood, M. S. A., Košler, J., Gehrels, G., Jackson, S. E., McLean, N. M., Paton, C., Pearson, N. J., Sircombe, K., Sylvester, P., Vermeesch, P., Bowring, J. F., Condon, D. J., and Schoene, B., 2016, Community-Derived Standards for LA-ICP-MS U-(Th-)Pb Geochronology – Uncertainty Propagation, Age Interpretation and Data Reporting: Geostandards and Geoanalytical Research, v. 40, no. 3, p. 311-332, https://doi.org/10.1111/j.1751-908X.2016.00379.x

Jochum, K. P., Weis, U., Stoll, B., Kuzmin, D., Yang, Q., Raczek, I., Jacob, D. E., Stracke, A., Birbaum, K., Frick, D. A., Günther, D., and Enzweiler, J., 2011, Determination of Reference Values for NIST SRM 610–617 Glasses Following ISO Guidelines: Geostandards and Geoanalytical Research, v. 35, no. 4, p. 397-429, https://doi.org/10.1111/j.1751-

908X.2011.00120.x

Ludwig, K. R., 2012, User's manual for Isoplot 3.75: Berkley Geochronology Center Special Publication, v. 5, p. 1-75,

Mikkelsen, L., 2020, Tracing Sveconorwegian magmatic provinces in the Nordfjord region, western Norway: A Zircon U-Pb geochronological and petrological study: University of Bergen.

Parrish, R. R., Parrish, C. M., and Lasalle, S., 2018, Vein calcite dating reveals Pyrenean orogen as cause of Paleogene deformation in southern England: Journal of the Geological Society, v. 175, no. 3, p. 425-442, 10.1144/jgs2017-107

Paton, C., Hellstrom, J., Paul, B., Woodhead, J., and Hergt, J., 2011, Iolite: Freeware for the visualisation and processing of mass spectrometric data: Journal of Analytical Atomic Spectrometry, v. 26, no. 12, p. 2508-2518, https://doi.org/10.1039/C1JA10172B

Paton, C., Woodhead, J. D., Hellstrom, J. C., Hergt, J. M., Greig, A., and Maas, R., 2010, Improved laser ablation U-Pb zircon geochronology through robust downhole fractionation correction: Geochemistry, Geophysics, Geosystems, v. 11, no. 3, p. Q0AA06, https://doi.org/10.1029/2009gc002618

Petrus, J. A., Chew, D. M., Leybourne, M. I., and Kamber, B. S., 2017, A new approach to laser-ablation inductively-coupled-plasma mass-spectrometry (LA-ICP-MS) using the flexible map interrogation tool ‘Monocle’: Chemical Geology, v. 463, p. 76-93, http://dx.doi.org/10.1016/j.chemgeo.2017.04.027

Petrus, J. A., and Kamber, B. S., 2012, VizualAge: A Novel Approach to Laser Ablation ICP-MS U-Pb Geochronology Data Reduction: Geostandards and Geoanalytical Research, v. 36, no. 3, p. 247-270, https://doi.org/10.1111/j.1751-908X.2012.00158.x

Roberts, N. M. W., Drost, K., Horstwood, M. S. A., Condon, D. J., Chew, D., Drake, H., Milodowski, A. E., McLean, N. M., Smye, A. J., Walker, R. J., Haslam, R., Hodson, K., Imber, J., Beaudoin, N., and Lee, J. K., 2020, Laser ablation inductively coupled plasma mass spectrometry (LA-ICP-MS) U–Pb carbonate geochronology: strategies, progress, and limitations: Geochronology, v. 2, no. 1, p. 33-61, https://doi.org/10.5194/gchron-2-33-2020

Roberts, N. M. W., Rasbury, E. T., Parrish, R. R., Smith, C. J., Horstwood, M. S. A., and Condon, D. J., 2017, A calcite reference material for LA-ICP-MS U-Pb geochronology: Geochemistry, Geophysics, Geosystems, v. 18, no. 7, p. 2807-2814, https://doi.org/10.1002/2016GC006784

Stacey, J. S., and Kramers, J. D., 1975, Approximation of terrestrial lead isotope evolution by a two-stage model: Earth and Planetary Science Letters, v. 26, no. 2, p. 207-221, https://doi.org/10.1016/0012-821X(75)90088-6

Woodhead, J. D., and Hergt, J. M., 2001, Strontium, Neodymium and Lead Isotope Analyses of NIST Glass Certified Reference Materials: SRM 610, 612, 614: Geostandards Newsletter, v. 25, no. 2-3, p. 261-266, https://doi.org/10.1111/j.1751-908X.2001.tb00601.x
